# Perspectives of people with aphasia post-stroke towards personal recovery and living successfully: A systematic review and thematic synthesis

**DOI:** 10.1371/journal.pone.0214200

**Published:** 2019-03-22

**Authors:** Molly Manning, Anne MacFarlane, Anne Hickey, Sue Franklin

**Affiliations:** 1 School of Allied Health, Faculty of Education and Health Sciences, University of Limerick, Limerick, Republic of Ireland; 2 Graduate Entry Medical School (GEMS), Faculty of Education and Health Sciences and Health Research Institute, University of Limerick, Limerick, Republic of Ireland; 3 Dept Psychology, Royal College of Surgeons in Ireland, Dublin, Republic of Ireland; Università degli Studi di Perugia, ITALY

## Abstract

**Background:**

There is increased focus on supporting people with chronic conditions to live well via person-centred, integrated care. There is a growing body of qualitative literature examining the insider perspectives of people with post-stroke aphasia (PWA) on topics relating to personal recovery and living successfully (PR-LS). To date no synthesis has been conducted examining both internal and external, structural influences on living well. In this study, we aimed to advance theoretical understanding of how best to promote and support PR-LS by integrating the perspectives of PWA on a wide range of topics relating to PR-LS. This is essential for planning and delivering quality care.

**Methods and findings:**

We conducted a systematic review, following PRISMA guidelines, and thematic synthesis. Following a search of 7 electronic databases, 31 articles were included and critically appraised using predetermined criteria. Inductive and iterative analysis generated 5 analytical themes about promoting PR-LS. Aphasia occurs in the context of a wider social network that provides valued support and social companionship and has its own need for formal support. PWA want to make a positive contribution to society. The participation of PWA is facilitated by enabling environments and opportunities. PWA benefit from access to a flexible, responsive, life-relevant range of services in the long-term post-stroke. Accessible information and collaborative interactions with aphasia-aware healthcare professionals empower PWA to take charge of their condition and to navigate the health system.

**Conclusion:**

The findings highlight the need to consider wider attitudinal and structural influences on living well. PR-LS are promoted via responsive, long-term support for PWA, friends and family, and opportunities to participate autonomously and contribute to the community. Shortcomings in the quality of the existing evidence base must be addressed in future studies to ensure that PWA are meaningfully included in research and service development initiatives.

**Systematic review registration:**

International Prospective Register of Systematic Reviews PROSPERO 2017: CRD42017056110.

## Introduction

Stroke is a leading cause of chronic disability with increasing absolute incidence and global burden [1, 2]. Many people live with multiple physical, psychological, social and financial consequences, reporting unmet clinical and social needs many years post-stroke [3, 4]. About one-third are affected by aphasia, a communication impairment affecting speaking, understanding, reading and writing, and associated with poorer outcomes and long-term residual difficulties including morbidity, hospital length of stay, quality of life, social networks and return to work [5–10]. A lack of high-quality guidance about the optimum approach to rehabilitation may impact on the quality and consistency of care delivery [11–14].

The lived experience and personal significance of chronic conditions is individual and dynamic, and people learn to cope, adapt, manage and live well in the context of illness in diverse ways [15]. There is increasing focus on the provision of a continuum of person-centred, integrated care coordinated across different health and related settings according to people’s needs throughout the life course [16, 17]. This type of care incorporates the perspectives of individuals, families, caregivers and communities, and empowers patients to play an active role in shaping health policy and services [17]. This is in line with growing emphasis on shared decision-making and guidelines that are underpinned by patient involvement and qualitative evidence about experiences, needs and values [18, 19]. Recovery from chronic conditions has traditionally been characterised from a purely biomedical point of view, emphasising the identification and treatment of standard disease processes [20]. Contemporary approaches such as self-management, the personal or social recovery model and the successful aging paradigm offer potential alternatives for supporting people to live well and to achieve personally meaningful outcomes.

Increased focus on supporting people to play an active role in managing personal health is reflected in a rise in self-management support initiatives such as the Stanford Chronic Disease Self-Management Course, the Expert Patients Programme in the United Kingdom and various condition-specific interventions [21–29]. Self-management support is designed to empower patients to monitor, manage and cope with their conditions targeting outcomes such as health-related quality of life, confidence, self-efficacy, health protective behaviours and access to health services [30]. The self-management paradigm has been criticised for failing to consider individual differences in capacity (skills, experiences, energy) and ability to cope with chronic conditions, and the influence of external social structures (social support, health services and economic and political factors) on wellbeing and ability to engage in self-care or self-management activities [31–33]. Self-management interventions may inadvertently cause patients to experience ‘treatment burden’ and associated adverse outcomes in response to self-management activities imposed by health providers [31, 34, 35]. The implications of delegating treatment work should be considered in terms of an individual’s capacity and relational resources and the underlying inequalities in the way that services may mobilise and support these resources [31, 32]. Interventions should fit into people’s lives and target person-centred outcomes such as life goals and wellbeing [31].

Personal recovery, a guiding vision in mental health service delivery, recognises both individual and structural influences on coping and living well. It is linked with empowering people to understand and manage their condition and symptoms and to fulfil life goals. It is also concerned with the removal of social, attitudinal and economic barriers to meaningful social inclusion and participation and with mobilising relational resources in the social network and community [36–39]. Personal recovery is individually-defined, ongoing and non-linear and can take place within or without the mental health system [36–38]. This focus on an individual’s health, wellness and resources in the presence or absence of medical illness contrasts with biomedical conceptualisations of recovery, more concerned with impairment and deficit [37–40].

Within gerontology, successful aging addresses what it means to age well or successfully. Early definitions were largely researcher-led with a biomedical-focus, emphasising freedom from disability and active engagement with life. Incorporating the views of older people has led to a different conceptualisation of successful aging more concerned with psychosocial “adaptation, meaningfulness and connection” [41]. It is now generally acknowledged that successful aging is possible for people with disabilities and chronic disease, however this area remains understudied [42]. Despite these strengths, the successful aging paradigm has been criticised for failing to consider the effect of poverty, early life influences, pre-existing disability and wider social and cultural factors and public policy on a person’s potential for aging well [42, 43]. The term ‘success’ has been described as polarising in creating successful and unsuccessful aging classes [43]. In emphasising individual effort, agency and responsibility, the idea of ‘success’ may inherently lay the blame on individuals when they do not age successfully, creating a risk of neglect and lack of support [42, 43].

In recent years, a small number of studies focused explicitly on the concept of living well or successfully with aphasia. Coined from the successful aging paradigm, living successfully with aphasia is broadly taken to mean “something beyond coping…participating in life as fully as one chooses” [44]. The concept was initially explored in a series of small, exploratory qualitative studies in 2006. Holland presented the stories of 3 exemplars of people considered to be living successfully with aphasia post-stroke [44]. Boles attempted to describe ‘success’ in living with aphasia through interviews with 2 couples who self-identified as living successfully [45]. Hinckley conducted secondary analysis of published accounts of people with post-stroke aphasia (PWA) to understand what it takes to live successfully with stroke and aphasia [46]. Cruice and colleagues selected the accounts of 4 older PWA and described their perspectives about quality of life, making suggestions for successful living [47].

Most of these studies were informed by researcher-defined exemplars of living successfully with aphasia. Subsequent studies, however, have attempted to explore this concept from the insider perspective by interviewing larger and more diverse samples of PWA about the meaning of living successfully and the factors influencing it [48–50]. Brown and colleagues interviewed 25 PWA a minimum of 2 years post-onset and illustrated the highly individualised, complex nature of living successfully comprising 4 core themes: doing things, meaningful relationships, striving for a positive way of life and communication [50]. In a later study, they integrated these and findings of other interview studies with family members and SLT’s generating 7 overarching themes: participation, meaningful relationships, support, communication, positivity, independence and living successfully as a journey over time [51]. Grohn and colleagues conducted a longitudinal study examining the perspectives of 15 PWA towards factors facilitating living successfully at 3, 6, 9 and 12 months post-stroke. Their analysis illuminated the dynamic and complex nature of living successfully as people’s perspectives change over time. Actively moving forward, perceived communication improvement, engagement in activities, social support and maintaining positivity were all important components [48, 49].

Living successfully with aphasia research is complemented by a growing body of qualitative research examining the lived psychosocial experiences of aphasia via inter-related topics like participation, community integration, life roles, coping, adjustment and quality of life [47, 52–65]. However, there is a lack of conceptual clarity about how living successfully with aphasia fits with these related areas. Additionally, living successfully with aphasia research has predominantly focused on individual processes of adaptation and coping at the expense of examining wider structural influences on wellbeing and ability to engage in self-care activities.

Qualitative evidence synthesis methods enable the findings of individual qualitative studies to be integrated in order to advance conceptual understanding and to inform health service delivery, clinical guidelines and the development and implementation of complex interventions [66–68]. Two prior qualitative evidence syntheses have explicitly included the perspectives of PWA. These examined the concept of treatment burden in stroke management and the long-term care needs of people with post-stroke communication difficulties (including aphasia, dysarthria and apraxia of speech) from the patient perspective [34, 69]. The latter review highlighted the long-term nature of the psychosocial support needs of people with post-stroke communication difficulties, identifying 5 key aspects which the authors suggested might be addressed through future self-management interventions: managing everyday communication outside the home; creating a meaningful role; creating and maintaining a support network; and taking control and actively moving forward with life [69]. The review focused on the individual processes of coming to terms with and adapting to communication disability rather than wider structural and equity issues to do with living well and accessing support.

To avoid treatment burden and other pitfalls of individualistic self-management approaches described earlier, the scope of living successfully with aphasia must be refined so that it is more incisive and analytical. To the best of our knowledge, to date, no qualitative synthesis has examined and integrated the range of concepts and topics relating to and influencing personal recovery and living successfully (PR-LS) from the perspectives of PWA.

In this study, we have synthesised prior qualitative interview studies in which PWA were asked about topics relating to internal and external influences on PR-LS. The review was interpretive and aimed to advance conceptual understanding to guide planned primary studies. The objective was to systematically identify and synthesise prior qualitative research examining the perspectives of adults with aphasia post-stroke towards PR-LS and related concepts. The research questions were:

1. What do PR-LS mean to PWA?
2. Who or what facilitates PR-LS according to PWA?
3. What gets in the way of PR-LS according to PWA?
4. What ideas do PWA have about what could or should be done to promote PR-LS?

## Methods

The protocol for this study was registered with the International Prospective Register of Systematic Reviews (PROSPERO) CRD42017056110 (S1 Text) [70]. This review is reported following the Enhancing Transparency in Reporting the Synthesis of Qualitative Research (ENTREQ) and Preferred Reporting Items for Systematic Reviews and Meta-analyses (PRISMA) statements (S1 and S2 Checklist) [71, 72]. We followed the thematic synthesis method described by Thomas and Harden, which enables the findings of individual qualitative studies to be inductively interpreted, synthesised and abstracted in response to the research questions in order to generate new insights and hypotheses beyond the primary studies [73]. The review team comprised 2 SLT researchers (MM, SF) a social scientist (AM) and a health psychologist (AH).

### Search strategy

A comprehensive search strategy was devised with an information retrieval expert in order to locate all potentially relevant studies (S1 Table) [74]. Preliminary scoping searches of personal reference manager files, Google Scholar and EMBASE were conducted using the ‘berry-picking’ method to source key articles, search terms and concepts. Search terms and concepts were derived from the research objective and organised according to the SPIDER framework for retrieving qualitative literature as follows: qualitative interview or focus group studies (Design), published in peer-reviewed journals (Research type) examining the perspectives (Evaluation) of adults aged 18 years and over with post-stroke aphasia (Sample) towards topics relating to PR-LS (Phenomenon of Interest) [75]. We designed a single line search syntax (Sample AND (Design OR Evaluation OR Research type) AND Phenomenon of Interest) in EMBASE using free text terms and Emtree headings and synonyms before optimising for Medline (Ovid), PubMed, Web-of-Science, Scopus, CINAHL and PsycINFO (EBSCO) [74]. No date or language restrictions were imposed. The electronic databases were searched in January and March 2017. Search results were downloaded to EndNote X7 and duplicates were removed. This was supplemented with hand-searching of the journal Aphasiology, reference and citation tracking of included studies and consulting with experts.

### Study selection

The first author and a second researcher independently screened title / abstract and full text articles against the inclusion and exclusion criteria (S2 Text). Discrepancies were resolved through discussion. Mixed methods studies and studies examining the perspectives of mixed groups were included if it was possible to extract data obtained through qualitative interviews with PWA. Studies describing experiences and perspectives in an experimental situation (e.g. developing or evaluating a specific tool or intervention) and studies with a specific focus on (social) communication or information needs were excluded.

### Critical appraisal

The first and third authors independently critically appraised 5 randomly selected studies using an instrument adapted from Rees et al [76]. This instrument provided clear guidance for producing weighted trustworthiness (reliability) and overall usefulness scores, which meant that quality was appraised in the context of the aims of both primary studies and the present synthesis and considered the extent to which the views of PWA were represented in the data extracted. The instrument was refined following discussion of initial appraisal results (S2 Table). The first author appraised the remaining studies. Studies were not excluded based on quality alone. The purpose was to examine the relative contribution of individual studies to the final interpretations and to inform subsequent discussion and analysis.

### Data extraction

The first author and a second researcher independently extracted data from 6 randomly selected articles using a pre-prepared data extraction instrument. Following discussion, the instrument was refined (S3 Table). The first author extracted data from the remaining articles in consultation with the final author. All text from the ‘findings/results’ section relating to the experiences, preferences and perspectives of PWA discovered through qualitative interviews and/or focus group discussions was extracted verbatim and uploaded to NVivo 11 [77]. Additional text appearing in other sections that described the findings differently was also extracted [73].

### Data analysis and synthesis

We followed the 3 stages of thematic synthesis, first applying line-by-line coding to inductively develop initial descriptive codes [73]. Next, we applied axial coding, looking for similarities and differences among the codes to create descriptive codes. Much of the data represented in the coding were opposing, and so grouping themes according to barriers and enablers was often a useful and meaningful strategy in order to make sense of the large amount of data and codes generated. The first and second authors independently open-coded three of the articles and met to discuss differences in approaches taken. The first author completed the open and axial coding, regularly consulting with the other authors. A narrative summary of the descriptive themes was circulated to all authors for their input and a final version was agreed.

The third stage involved further interpretation and abstraction of the data to generate analytical themes about promoting PR-LS. This process of ‘going beyond’ the original studies is akin to generating third-order interpretations within meta-ethnography [73, 78]. To maintain both analytical transparency and ‘richness’ of content, the first author developed a set of barrier and enabler statements summarising the content of the descriptive themes. These were grouped into 5 analytical themes. Over a series of data analysis workshops with all authors, the themes were further refined and abstracted such that they both described and surpassed all of the initial descriptive themes and barrier and enabler statements (73).

## Results

### Study selection

**Fig 1:**
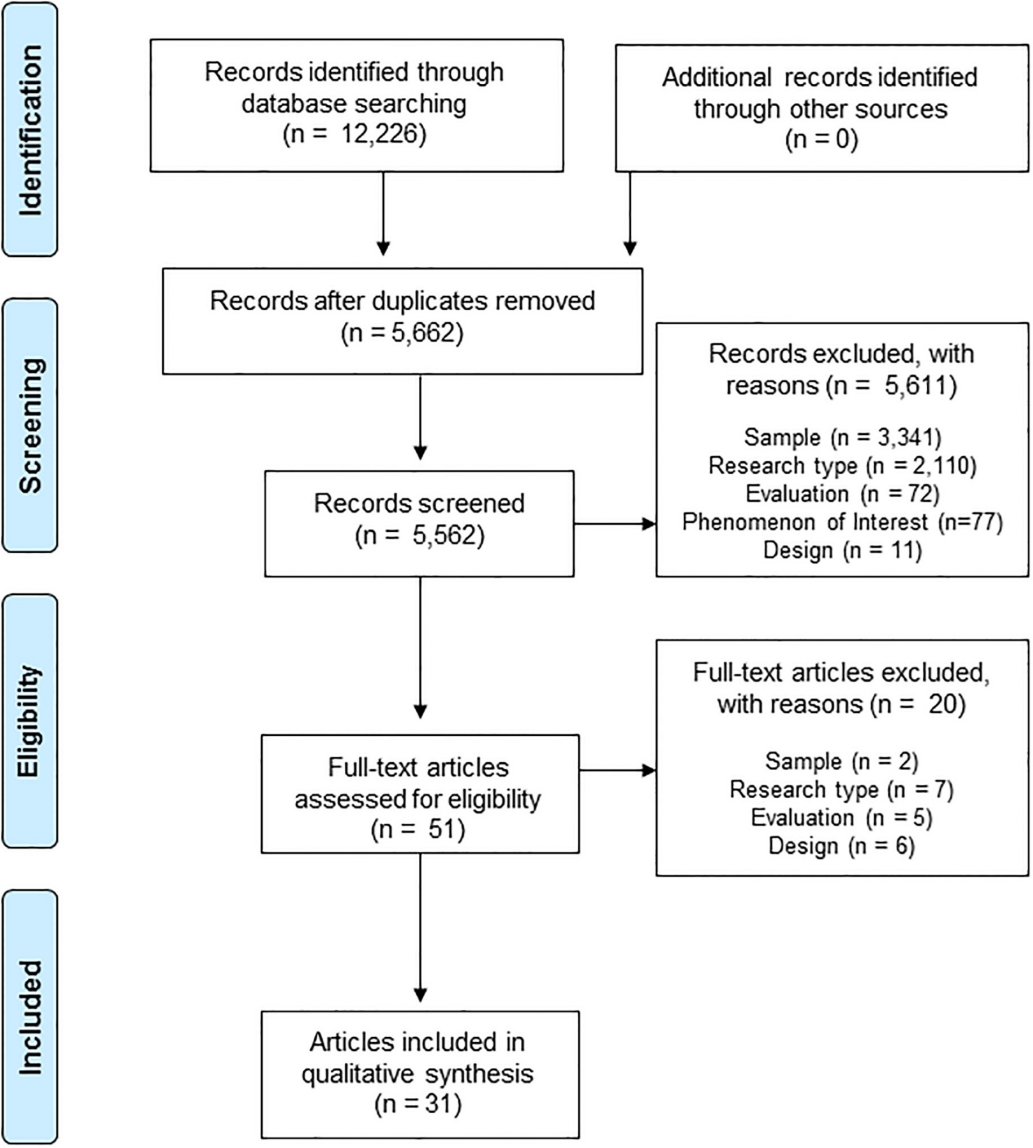
PRISMA flow diagram [72].

The electronic database search yielded 12,226 citations. A total of 5,662 titles/abstracts were screened following de-duplication. A total of 51 full text articles were retrieved and assessed for eligibility. Of these, 20 articles were excluded as they did not meet our inclusion criteria: 2 did not include any PWA because of stroke; 5 did not describe qualitative methods of data collection and / or analysis and 1 study did not use qualitative interviews; it was not possible to extract PWA contributions from 5 interview studies with mixed participant groups; and 7 were not peer-reviewed journal articles. We contacted the author of one potentially relevant citation in the form of a dissertation; however, no peer-reviewed article was available. A total of 31 studies were included in the thematic synthesis, each sourced via electronic database searching (Fig 1).

### Characteristics of included articles

The included articles described the perspectives of 350 PWA from 10 high-income countries, all published in English (S4 Table) [79]. Research from 24 separate studies was presented including both primary (n = 27) and secondary (n = 4) data analyses. These studies examined a diverse range of topics including: living successfully with aphasia (n = 3) [48–50]; quality of life (n = 2) [47, 80], participation, including community, civic and societal participation and experiences of volunteering [81–85] (n = 5); the lived experience and consequences of aphasia, including social role changes, engagement in everyday occupations, grief and loss issues, factors influencing recovery and hope (n = 11) [54, 55, 59, 64, 65, 86–91]; friendship (n = 2) [92, 93]; experiences of health and related services, including adverse events, collaborative goal-setting and discharge (n = 6) [94–99]; legal and access to justice issues (n = 1) [100]; and goals (n = 1) [101].

### Quality of included studies

Initial agreement on critical appraisal was 85%, increasing to 100% following discussion. Ten articles had high overall weighted reliability, demonstrating fairly thorough attempts at increasing rigour in sampling, data collection and analysis and grounding the findings in the data presented. The remaining articles had medium (n = 18) or low (n = 3) overall weighted reliability. Reasons for lower reliability scores included lack of specification of steps taken to increase rigour in sampling (n = 5), data collection (n = 3), analysis (n = 4) or fair/limited grounding of findings and interpretations in the data presented (n = 14). Just under half of the articles (n = 15) were rated highly useful in the context of the synthesis (weighted scores) considering the extent that the perspectives of PWA had been privileged and the breadth and depth of author interpretations. The remainder were of medium (n = 8) or low usefulness (n = 8), demonstrating a lack of breadth (n = 8) or depth (n = 9) of the findings, or that perspectives of participants had been privileged ‘somewhat’ (n = 14) or ‘a little’ (n = 7) (S5 Table).

### Analytical themes

We created 85 descriptive codes and 16 descriptive themes (S3 Text). A set of barrier and enabler statements was developed to summarise the content of the descriptive themes, whilst maintaining the breadth and richness of the underlying content (S4 Text). These were grouped and iteratively abstracted and refined to generate 5 analytical themes. These themes, described below, are interpretive constructs that go beyond the primary studies and generate new ideas about promoting PR-LS.

#### (1) Aphasia occurs in the context of a wider social network that provides valued support and social companionship and has its own need for formal support

Thirty articles contributed to this theme. Aphasia occurs in the context of a person's social network that is a valued source of social connection and support. PWA valued social ease, tolerance, acceptance and understanding from family and friends and not being treated differently than before the stroke [50, 89, 96, 101]. This was illustrated in the case of a mother of two school-aged children: “To them I’m just Mum … They don’t care [that I’ve got aphasia]” [50]. Friends and family provided emotional support, encouragement and help to stay positive, and practical help with household duties, activities of daily living, self-care, managing finances, social and community participation, communication support, transport and support in hospital or with therapy activities [48–50, 81, 82, 92]. However, PWA across several studies experienced support as disempowering and had negative or ambivalent feelings of reduced autonomy, dependence, lack of privacy and loss of reciprocity or feeling like a burden on others [50, 82, 83, 85, 88, 89, 91, 92].

People wanted to be “able to contribute by doing things for their families or partners, for example shopping for the family, organising birthday parties for a son/daughter, or looking after a partner’s rose garden” [50]. Some had adapted to changed family roles, for example one man who became a stay-at-home parent while his wife went to work [82]. Feeling connected, maintaining relationship roles like parent or spouse and having good or meaningful relationships were important [50, 80, 83, 86, 87, 89]: “I think it’s really people … that make you feel successful” [50]. One woman had been uncertain whether she would be able to maintain custody of her children following her stroke. Seven years post-onset, having her children with her on a part-time basis and maintaining her role as a mother “was paramount to her concept of living successfully” [50].

At the same time, PWA experienced many negative changes to relationships and relationship roles including friendship loss and social isolation, lack of acceptance, loss of reciprocity and over-protection [50, 65, 81–83, 85, 92, 93]. Aphasia restricted ability to “maintain identities as the diverse, subtle purposes of communication (distracting an infant; reassuring a confused elderly parent; saying good-bye to a dying spouse) may be unachieved” [59]. One woman was frustrated by her inability to relate to her husband as before: “I have no ability to have a discussion with T. [her husband] or anything else like this because he can’t do … he can understand anymore, and isn’t worth it all in the outcome or anything else like that that I do anything about it” [90]. For many, parenting roles and relationships with children were negatively affected [59, 82, 85–87]. Parents of older children “encountered problems responding with producing the right kind of language for particular situations” for example “the 18-year-old about to depart to college, the 20-year-old who has become addicted to heroin or the adolescent who is being bullied at school” [59]. Some people spoke about how their children had turned away from them, pushed them out and/or viewed them as silly [59]. One man described how he “could communicate fairly easily with his boys when they were babies and toddlers, reading them simple stories and joining in their chatter”, however aphasia “began to weaken his sense of identity as a father” as they grew older and their “demands became more sophisticated” [59].

Several studies highlighted a lack of inclusion, support and information for family members from health and related services [82, 97, 99]. Excluding spouses, who could provide crucial communication and memory support to PWA, from ward rounds and medical consultations resulted in distress and missed information [97]. A lack of support and training for family members was linked with negative relationship changes and tensions post-stroke as one man’s spouse “did not seem to understand how to assist him” [82]. Access to aphasia information and training for the friends and families of PWA is necessary to increase understanding and acceptance of aphasia, to support the maintenance of friendships, relationships and relationship roles, and to improve the acceptability of social support by the social network.

#### (2) PWA want to make a positive contribution to society

All 31 articles contributed to this theme. Participation and autonomy were reduced due to aphasia and other impacts of stroke (physical, cognitive and psychological) [47, 50, 54, 65, 80–83, 85–87, 89, 90, 94, 95, 101], concomitant health conditions [47, 50, 80, 86, 87, 101], reduced ability to get around the local environment independently [48, 50, 59, 65, 80, 82, 88, 92, 94, 99], not living in one’s own home [47, 80, 91, 94, 95, 101], reduced income and /or ability to manage finances [59, 82, 83] and avoidance of speaking and social situations [47, 48, 50, 59, 65, 81–83, 85, 88–90, 92, 93, 95]. Restricted ability to take part in and to enjoy previous activities impacted negatively on well-being, hope, quality of life and sense of self [47–50, 54, 65, 80, 89, 90, 95, 101].

At the same time, PWA across several studies felt that it was important to do “as much as you can” and to have an active life at a satisfying level [47, 48, 50, 80]. They engaged in a range of personally meaningful activities, contrasting ‘doing things’ with “sitting home doing little” [49, 50, 59, 81, 84, 85, 87, 92, 98, 101]. This was seen as important for distracting and stimulating the mind, alleviating negative emotions, re-establishing routine and gaining a sense of purpose, autonomy and control [47–50, 81, 90, 101].

Some had successfully returned to previously valued activities including work, paid and voluntary, driving and education [50]. Some were trying new activities to replace lost ones or adapting previous activities to adjust to their new abilities [47]. The pursuit of new activities was potentially enriching and contributed towards sense of self [47]. Through doing and participation, people updated their perceptions of their capacity, regained confidence, positive self-esteem and feelings of usefulness and worked towards independent engagement in higher levels of activity [49, 81, 89]. Developing new skills, finding new activities and ways to help others was part of self-growth and adapting to a new reality [90].

PWA wanted to be recognised as useful, contributing members of their communities for example by returning to paid or voluntary work and helping others. People across several studies had experienced loss of employment, which was interconnected with sense of self and self-identity, personal finances and social participation, and which resulted in boredom, isolation and desire to return to work [48, 49, 55, 59, 82, 86, 89, 99, 101]. Younger people were particularly aware of loss of career and held strong desires to return to employment, sometimes volunteering if this was not possible [86, 94, 101]. People were “motivated by a desire for activity which did not centre on the home…and by wanting to effect change for themselves and the populations they represented, which they saw as disadvantaged or excluded from society” [81, 84, 85, 101]. One woman spoke about her “desire to give back to other people with her condition…she wanted to teach them that many things are possible, that things do get better, and that their lives are not over. She aspired to be a source of empathy and understanding for people with aphasia who feel misunderstood” [90]. Some people felt qualified to support others going through similar experiences providing inspiration or advice to others and sharing experiences [48, 81, 86, 90]. They also recognised that they could help themselves through helping others [84].

#### (3) The participation of PWA is facilitated by opportunities and supportive, enabling environments

Twenty-five articles contributed to this theme. Participation was facilitated through opportunities to socialise and meet others [48, 64, 82, 83, 85, 87, 92, 96], increased public awareness of aphasia (50, 81, 83, 84, 101), skilled and supportive conversation partners [49, 65, 81–83, 85, 89], and policies and procedures to support autonomous participation in the workplace [59, 81, 82] and in public and commercial services and facilities [49, 82, 83].

PWA valued opportunities for social contact in the community including opportunities to meet and socialise with others with stroke and aphasia in support and therapy groups [48, 83, 92, 96]: “it’s real good, because you can … you can … well you can talk to them. And you know you talk and you’re getting through. And talking helps you. It helps you” [48]. People attending aphasia organisations felt “welcomed, protected, included and unconcerned about being judged by others” and had the opportunity to share and make sense of experiences, receive support and meet new friends [82]. However, not everybody felt positively about attending stroke and/or aphasia groups: one man was worried “about not having the skills to communicate” with other group members; another “found it distressing at times to see the negative consequences of stroke and aphasia in others” [48]. Other PWA attended other non-aphasia-specific disability groups and community-based interest groups [64, 85, 87].

PWA referenced a general lack of knowledge about post-stroke aphasia and the many attitudinal and structural barriers to participation they experienced, including discrimination, prejudice and disabling attitudes and restricted access to the work environment and to commercial services [50, 59, 81, 83–85, 89, 92, 98, 100]. ‘Doing things’ did not necessarily make PWA feel more integrated and many described negative experiences of participating in community life, which was frustrating, depressing and tiring [83–85]. Return to work or volunteering was negatively experienced when there was a lack of appropriate support or a mismatch of expected duties and skillsets. Many PWA felt bypassed, ignored, pushed out or shunned, e.g., “When I go to a restaurant or a pub I get ignored totally”, or perceived as “crazy”, “silly”, an “idiot” and/or not useful, contributing members of the community [50, 59, 81, 83, 85, 89].

PWA felt that improved public knowledge of the condition and lived experience of aphasia and communication strategies were important for removing barriers to their participation [50, 81, 83, 84, 101]. Awareness, tolerance and understanding from others was valued [82, 83]: “When I say in a shop: ‘I have been sick. I had a stroke.’ People there: ‘Take your time ma’am. We’ll help you.’ That’s nice that” [82]. Specific characteristics of skilled, facilitative communication partners including “willingness, skills and knowledge” were described in several studies [49, 65, 81–83, 85, 89]. Being open about one’s difficulties, actively seeking support and patience with communication, and asserting oneself in order to be heard and to confront disabling attitudes were also seen as important [49, 50, 55, 59, 81–84, 92, 93, 97, 98, 100, 101]. Factors that supported confidence to initially engage in voluntary and paid work included others’ awareness of aphasia, opportunity to retrain, modified working hours and accessible events and meetings, for example meeting the chair in advance to discuss agenda items [59, 81, 82]. In commercial and non-health public utilities and services, including banking, legal and insurance transactions, PWA were supported to participate autonomously when provided with accessible, non-complex written and verbal information, quiet environments, support with purchasing and handling money, automatic banking procedures that minimised the need for verbal or written communication and additional consultation time and/or communication support, for example having extra time for communication in government offices, on buses and in community places in general, and assistance with completing forms and paperwork [49, 82, 83]. One author commented that changing many features of contemporary lifestyle and job demands may not be feasible nor straightforward [59].

#### (4) PWA benefit from access to a flexible, responsive, life-relevant range of services in the long-term post-stroke

Twenty-nine articles contributed to this theme. People’s experiences highlighted a need for access to a range of health and social care services in the long-term to support ongoing self-directed recovery and self-growth and that “recognise the person as a whole and not just someone who has aphasia” [81]. At the same time, a lack of follow-up therapy services post-discharge from hospital was referenced across several studies [87, 97, 100]. Levels of satisfaction with health care were influenced by amount of therapy provided, swiftness of service provision and perceived amount of support received at specific times including discharge and transition home [96].

The need for formal emotional support including self-help groups (with separate groups for spouses), medical management of depression and access to psychology, to help people to cope and come to terms with multiple losses and the negative psychosocial consequences of stroke and aphasia was referenced in several studies [48, 50, 85, 90]. One woman spoke about psychological support in the first-year post stroke: “I think, yeah, going to see [psychologist] has been huge … And it has been good. Well the first day I went I thought no, this is not for me. And I went back the following week and yeah, no, I’ve been going back every three weeks now” [48]. At the same time, the insufficiency of support for dealing with losses, particularly before discharge, was evident. In one study, one participant alone had received satisfactory counselling [82].

Coming to terms with and accepting aphasia, renegotiating identity, cultivating positivity and actively directing recovery and self-growth require long-term perseverance and effort. The importance of formal therapy “to help maintain active engagement in the recovery process” and to provide “ideas and resources” in the long-term was clear [48]. At the same time, many PWA had experienced financial, geographical, informational and attitudinal barriers to accessing services (59, 83, 94, 96). Positive experiences of language rehabilitation were common [48–50, 91, 94]: “and the biggest thing that has helped me…the speech therapy session” [50]. However, the need for additional SLT resources and rehabilitation support in hospital was observed [82, 100].

Levels of satisfaction with the timing and frequency of therapy varied: some people would have preferred intensive therapy later in their recovery and others wanted flexibility to “dip in and out of therapy depending on their health and energy” [94]. One study highlighted the need for life-relevant therapy that lasted longer and that met people’s needs “at different stages of recovery” [101]. Therapy was appraised holistically in relation to overall health and broader life context. People were differentially concerned about post-stroke aphasia relative to physical impairments or comorbid health conditions [50, 86, 87, 101]. Some people were in “social, emotional and financial turmoil, and the therapies they experienced seem to take little account of these major upheavals in their lives” [59]. A specific lack of support in the long-term for relatively younger PWA, for whom there may be specific challenges in terms of parenting roles and reduced income, was highlighted [94, 95]. PWA across several studies found the content of language therapy too theoretical, difficult, patronising or irrelevant to their needs and this was linked with patient-led discharge and disinterest in further support [82, 86, 94, 96, 101]. One man had found SLT hard and made him feel useless “like little kids an’ that do there, you know?” [86]. One study highlighted potential benefits of involving PWA in the planning and running of acute and post-acute services including: assisting staff to do a better job by learning about lived experiences of aphasia; and providing other PWA with ideas for long-term support once therapy had stopped [81].

PWA accessed community health clinics, community organisations, home care/assistance programmes, caregivers and community nurses for support with activities like household chores, using the telephone and paperwork [50, 82, 86, 87, 90]. One participant stressed the importance of actively seeking out assistance: “if you don’t reach out, you get nothing; you get to stay alone sitting in your corner [82]. One participant was content and satisfied at moving into a supported unit in a retirement village with her husband, who also had aphasia, and so she could “easily call for support or care” [47]. Another felt lucky to be supported to live alone in her own house with a nursing service each morning and support with cooking and ironing each afternoon [50].

#### (5) Accessible information and collaborative interactions with aphasia-aware healthcare professionals empower PWA to navigate the health system and direct their own recovery

All 31 articles contributed to this theme. PWA wanted information about aphasia and treatment options to better understand and explain their condition to others, to organise and access services and to be actively involved in decisions about their care [99, 101]. For some, knowing recovery would take time fostered optimism [49], whereas for others, negative prognostic information disrupted hope, positivity and engagement with rehabilitation [50, 94, 96, 98]. A lack of access to information was highlighted: “but we search and search and come up [empty]” [50]. This further compounded uncertainty about prognosis and the degree to which PWA “felt able to influence the course of it” [83].

The value of positive relationships and interactions with healthcare professionals, including SLT’s, who were knowledgeable about aphasia and supporting communication needs was highlighted across several studies [49, 50, 82, 83, 96, 99, 101]: “at least I had someone to go and talk to about things” [96]. PWA appreciated being “seen and heard”, being spoken to directly and treated as equal and intelligent [96, 99]. At the same time, PWA across several studies described inaccessible encounters with medical professionals [82, 97, 101] and were frustrated, disappointed and irritated by being spoken over [94, 99, 100]: “What is the result of that test? Yes, you can explain that to my mother. But what about me? It’s my stroke and I am a professional so tell me” [94]. A lack of knowledge and training on the part of health professionals was disempowering: “the strength of feeling appeared to stem from negative experiences caused by interaction with people who lacked appropriate knowledge of aphasia or its effects” [81, 83]. Indeed, aphasia increased vulnerability and potential for adverse events in hospital and rehabilitation settings. One woman who felt “ashamed” at being showered by a male nurse had been unable to express this to anyone apart from her husband [87]. Another was distressed and angry at “wetting the floor” as she had not been able to communicate her basic care needs to a nurse [97]. Hemsley and colleagues observed that “although most undesirable events reported by the participants were described as being non-harmful, the manner of their telling and the detail recalled…suggested that the events were still highly salient, somewhat distressing, and important to the participants” [97].

Levels of involvement in clinical decision-making, goal-setting and therapy activities and responsiveness to patient feedback influenced levels of satisfaction and dissatisfaction with healthcare [96]. There were individual differences in people’s desired level of participation, from those who trusted the clinician as expert to make decisions in their best interests to those who wanted to be involved and who may have been dissatisfied at the lack of collaboration [96, 99, 101]. Some people accepted that collaboration could be difficult at certain stages, including in the initial acute and post-acute stages when language was more severely affected, during which they needed next-of-kin support [99]. Poor communication or vagueness regarding the timeframe of therapy, discharge decisions and the availability of further community supports (and suitability for these supports) had important implications for people’s capacity to manage and direct their ongoing recovery [82, 99]. Some PWA thought their difficulties were too mild/severe to be eligible for further support and/or did not know “what was a reasonable request” on discharge from therapy [82, 94, 99]. Others did not want to jeopardise the therapeutic relationship or to waste the therapist's time by requesting more therapy, questioning decisions or overly directing goal-setting [94, 99].

## Discussion

To the best of our knowledge, this is the first qualitative evidence synthesis of the perspectives of people with post-stroke aphasia towards concepts relating to PR-LS. We synthesised 31 articles in which 350 PWA across 10 high-income countries were asked about their views on a diverse range of topics and concepts including the lived experience and consequences of aphasia, experiences of health and related services, participation, living successfully and quality of life. The spread of articles over time was 21 years. More than half (n = 17) were published within 5 years prior to searching, reflecting growing activity in this area, indicating the timeliness of this synthesis.

### Summary of findings

First, relationships, relationship roles and social support were important. Negative relationship changes were common, perhaps fuelled by a lack of formal support and information for friends and family, who themselves experience significant change and losses post-stroke. Second, people wanted to be seen as useful, contributing members of their communities through activities such as returning to work, volunteering and helping others. Many stressed the importance of being proactive about participation and socialising and being open to doing things differently. Participation provided an opportunity to develop confidence and autonomy, learn skills, re-evaluate sense of self and progress to higher levels of activity. Third, participation was both hindered and enabled by external attitudes and structures. People felt that increasing awareness of aphasia and communication strategies among service providers and the general public would help support participation. Some felt that it was important to be open about their difficulties in order to gain support and to be assertive in response to discrimination. Fourth, coming to terms with aphasia and learning to live well required time, perseverance and access to responsive, flexible and person-centred support. However, people experienced multiple barriers to accessing appropriate services, particularly post-discharge. Fifth, aphasia rendered people vulnerable to adverse incidences and treatment burden. Accessible information and knowledgeable, trained healthcare professionals helped empower people to direct their recovery, organise care and collaborate in decisions about their treatment.

### Connections with literature

The analysis adds to the literature by presenting an expanded conceptualisation of PR-LS in the context of aphasia post-stroke. There is a growing body of qualitative literature examining the insider perspectives of PWA about the meaning of living well and related topics, but the literature tends to focus on a limited set of issues that relate to living successfully without due attention to wider structural influences recognising issues of equity in accessing support and completing self-care activities. We wanted to understand how best to support PWA to live well whilst acknowledging the diverse, individual and dynamic nature of this concept and without limiting the types of things that might matter. We therefore integrated a wide range of topics including the lived experience and consequences of aphasia, living successfully, participation and experiences of health and related services. As a function of the review design, the findings thus add to the literature by highlighting the need to consider wider attitudinal and structural influences on PR-LS.

The analysis complements life participation approaches to aphasia, which focus on a ‘broadening and refocusing of services’ to support people affected by aphasia in achieving their life-goals [102]. The findings support prior aphasia and stroke research highlighting the need for psychosocial support for coming to terms with loss, depression, grief and changes in life participation [61, 69, 102–105]. The analysis also supports prior recommendations for family-centred support, information and training in the context of aphasia [12, 106–114]. Access to information and to knowledgeable, aphasia-aware health professionals empowers PWA to take charge of their condition, collaborate on treatment decisions and navigate and advocate for further support services [12, 34, 106, 113, 115, 116]. This is important for minimising the treatment burden and significant stroke management demands experienced by people with stroke, for whom capacity to organise care, develop coping strategies and adhere to treatments is negatively impacted by fragmented services, a lack of continuity of care, poor information provision and poor communication with and between healthcare providers [34].

The analysis complements work demonstrating how participation is impacted by multiple environmental factors [117, 118] and calls for social inclusion and social philosophy approaches to aphasia [61, 119]. The analysis further supports a dynamic, multi-faceted conceptualisation of participation when construed from the insider perspective of people with disabilities (as opposed to classification systems such as the International Classification of Functioning, Disability and Health which emphasise disability and deficit) [117, 120]. This involves meaningful engagement, choice and control, personal and societal responsibilities, having an impact, access and opportunity, and social inclusion and membership [117]. The analysis also illustrates how younger PWA were particularly impacted in terms of loss of employment, income and changes to relationship roles including parenting, salient in the context of overall increasing numbers of people with stroke below 65 years [121] and supporting prior work around social participation and return to work [52, 122, 123].

### Methodological critique

In contrast with previous qualitative syntheses that included the perspectives of some PWA around treatment burden [34] and long-term support needs [69], we integrated topics relating to both internal and external influences on adaptation, personal recovery, life participation and living well in the context of aphasia, thus refining the conceptualisation of PR-LS and how best to promote living well with aphasia. The quality of the included studies was variable: just under one third (n = 10) and one-half (n = 15) of the papers respectively had high reliability and usefulness weighted ratings. Consequently, we conducted a sensitivity analysis to check the relative contribution of studies appraised as having low versus high reliability and usefulness [73]. We found that the higher quality studies had contributed most in terms of extracted data and in terms of the development of the analytical themes. We also attempted comparative analysis to explore possible patterns of association of the findings with participant characteristics, study country or health system; however contextual information was insufficient for this purpose.

A rigorous approach was taken throughout. Each stage had independent input from two researchers with frequent discussions and consensus meetings amongst the authors to develop the synthesis and interpretations. The team included just 2 SLT researchers (MM, SF), which might reflect a limitation in terms of maximising knowledge of the field and elements of therapy and treatment for PWA. However, the interdisciplinary nature of the review team, with a social scientist (AM) and health psychologist (AH) brought strengths including enhanced reflexivity during the qualitative analysis process [124] and encouraged explanation and exploration of issues for non-clinicians.

### Implications for future research

Responsive and relevant services must incorporate the perspectives of PWA [101] and their significant others [107]. There is a need for further research asking PWA and other stakeholders specifically about factors influencing living well in order to validate the refined conceptualisation of PR-LS in this synthesis. There is a need for studies that would implement and research PWA involvement in service development. Future studies should examine supports for PWA of working age and their families, including the needs of children and spouses and financial impacts. Finally, several shortcomings identified in the quality of the included studies must be addressed in future studies to ensure the meaningful inclusion of PWA in the research process and in-service development initiatives. These included: inadequate description of steps taken to include PWA in the interview process, for example through supported communication techniques; a lack of involvement of PWA in research design; and under-representation of the perspectives of people with severe aphasia.

## Conclusion

This synthesis advances conceptual understanding of PR-LS according to the perspectives of PWA post-stroke considering both internal and external factors to do with living well. Personal recovery and living successfully with aphasia are promoted via responsive, flexible and long-term support for PWA, their friends and family and the wider community, and through opportunities for people to participate autonomously and to make a genuine contribution to their communities. The themes need to be further validated with PWA and other stakeholders. Shortcomings in the quality of the existing evidence base must be addressed in future studies to ensure that PWA are meaningfully included in research and in-service development initiatives.

## Supporting information

S1 Text Review protocol. International Prospective Register of Systematic Reviews PROSPERO 2017. CRD42017056110.
(PDF)

S2 Text Study eligibility criteria. (PDF)

S3 Text Descriptive themes. (PDF)

S4 Text Barrier and enabler statements. (PDF)

S1 Checklist ENTREQ checklist. (PDF)

S2 Checklist PRISMA checklist. (PDF)

S1 Table Search strategy. (PDF)

S2 Table Critical appraisal instrument. (PDF)

S3 Table Data extraction instrument. (PDF)

S4 Table Summary of included studies. (PDF)

S5 Table Summary of critical appraisal. (PDF)
