# Supplementary material for: Perspectives of people with aphasia post-stroke towards personal recovery and living successfully: A systematic review and thematic synthesis
Source: PLoS One. 2019 Mar 22;14(3):e0214200. doi: 10.1371/journal.pone.0214200 (PMC6430359; doi:10.1371/journal.pone.0214200)
Supplement: S1 Text — International Prospective Register of Systematic Reviews PROSPERO 2017. CRD42017056110. (PDF) [file pone.0214200.s001.pdf]

Perspectives of people with aphasia post-stroke towards recovery, living successfully with aphasia and related constructs: a systematic review and qualitative thematic synthesis

Molly Manning, Anne Hickey, Anne MacFarlane, Sue Franklin

Citation

Molly Manning, Anne Hickey, Anne MacFarlane, Sue Franklin. Perspectives of people with aphasia post-stroke towards recovery, living successfully with aphasia and related constructs: a systematic review and qualitative thematic synthesis. PROSPERO 2017 CRD42017056110 Available from: http://www.crd.york.ac.uk/PROSPERO/display_record.php?ID=CRD42017056110

Review question

1. What are the perceptions and attitudes towards recovery and living successfully with aphasia of people with aphasia post-stroke? What do recovery and living successfully with aphasia mean to people with aphasia post-stroke?
2. Who or what do people with aphasia post-stroke think has helped them in their recovery and to live successfully with aphasia?
3. What do people with aphasia post-stroke think is unhelpful in terms of recovery and living successfully with aphasia?
4. What ideas do people with aphasia post-stroke have about what could or should be done to promote recovery and successful living with aphasia?

Searches

One reviewer (MM) developed a comprehensive topic-based search strategy with the support of an academic support librarian, Dr. Fintan Bracken, Research Services & Bibliometrics Librarian, University of Limerick, following the method outlined by Bramer and de Jonge (2015).
Databases searched: Embase, MEDLINE, Web of Science, Scopus, CINAHL and PsycINFO.
The search terms include free text and thesauri terms (Emtree, MeSH, CINAHL headings) relevant to the following key concepts, organised using the SPIDER tool for qualitative evidence synthesis (Cooke et al., 2012) as follows:
Sample: Aphasia.
Phenomenon of interest: Living successfully with aphasia, recovery, social participation, hope, friendship, coping, well-being, quality of life.
Design: Interview, focus group, grounded theory, constructivism, phenomenology.
Evaluation: Perspectives, experiences, preferences, opinions.
Research type: Qualitative.
The search syntax is of the form: S AND (D OR E OR R) AND PI. There is no language or date restriction.
Additional details can be found in the attached PDF document (link provided below).
Supplementary search strategy: Manual searching of key journals; reference and citation tracking of included studies; consulting with experts; scanning personal reference manager software; and checking reference lists of prior relevant reviews.

Search strategy

https://www.crd.york.ac.uk/PROSPEROFILES/56110_STRATEGY_20170027.pdf

Types of study to be included

Corresponds to Phenomenon of interest, Design & Research type (see Searches field).Inclusion criteria:1. Primary studies exploring the emic perspectives, experiences, preferences and / or opinions of adults with aphasia post-stroke on topics relating to recovery, living successfully and /or related constructs in the context of aphasia.2. Studies examining the perspectives of multiple populations will be included if it is possible to identify and extract the data obtained from people with aphasia post-stroke.3. The studies must employ

qualitative methods of data collection and analysis. Studies employing mixed methods will be eligible if it is possible to identify and extract the qualitative findings.4. The studies must be peer-reviewed and published.Exclusion criteria:1. Studies that do not examine perspectives of adults with aphasia post-stroke.2. Non-empirical articles including opinion pieces, commentaries, theoretical articles.3. Personal accounts that do not use qualitative methods of data collection and analysis. 4. Quantitative studies.5. Non-peer reviewed articles.6. Systematic reviews or syntheses. 7. Doctoral and Masters level theses or dissertations, and conference abstracts – however attempts will be made to source related, full-length articles published in peer-reviewed journals.

Condition or domain being studied

Aphasia is an impairment in speaking, understanding, reading and writing occurring in one third of people with stroke (Engelter et al., 2006). Health-related quality of life, return to work, morbidity and mortality are lower for people with aphasia than for other people with stroke (Cochrane et al., 2016; Hilari et al., 2012). People with aphasia are systematically excluded from the stroke literature (Brady et al., 2013).
There is a lack of consensus about what constitutes the optimum approach to rehabilitation (Clinical Centre for Research Excellence in Aphasia Rehabilitation, 2014). Processes of adaptation and recovery in the context of aphasia are not well understood, however findings from qualitative studies with people with aphasia post-stroke indicate that 'living successfully' with aphasia is a dynamic, individual and multi-faceted construct, facilitated in part by engagement in activities, meaningful relationships, social support, maintaining positivity, change in perception of self over time and perceived communication improvement (Grohn et al., 2014; Hinckley, 2006; Brown et al., 2010; Brown et al., 2012). To date there is no qualitative evidence synthesis of relevant qualitative literature regarding the emic perspectives of people with aphasia regarding what constitutes an optimal outcome / living successfully; what helps or doesn't help; what they want. There is also a lack of empirical research directly addressing the aims of this review. Research in this area is essential for delivering quality, integrated supports informed by people's needs and experiences.

Participants/population

Corresponds to Sample (see Searches field).
Inclusion criteria:
Adults aged 18 years and over with aphasia as a result of stroke.
Exclusion criteria:
1. People with aphasia of aetiologies other than stroke.
2. People with aphasia aged less than 18 years of age.
3. People who do not have aphasia as a result of a stroke.
4. No restrictions will be applied based on aphasia type or severity or any other demographic variable.

Intervention(s), exposure(s)

The objective of this review is to discover the perceptions of adults with aphasia post-stroke towards recovery, living successfully with aphasia and related constructs.

Comparator(s)/control

Not applicable.

Context

No further restrictions.

Primary outcome(s)

The aim is to systematically identify and synthesise empirical qualitative research explicitly examining the emic perspectives of people with aphasia post-stroke to explore:
1. What recovery, living successfully with aphasia (and related constructs) mean to people with aphasia;
2. Who or what facilitates recovery, living successfully with aphasia;
3. What is unhelpful in terms of recovery and living successfully with aphasia;
4. What ideas do people with aphasia have for what could or should be done to promote recovery and successful living with aphasia.

Secondary outcome(s)

A conceptual model of living successfully with aphasia which will inform further planned research.

Data extraction (selection and coding)

MM will download the electronic search results to EndNote and remove duplicates. MM will transfer titles and / or abstracts to NVivo for screening, data extraction and analysis following the approach described in Houghton et al. (2016). MM will be involved in study screening, data extraction, quality appraisal and analysis. Each stage will be checked independently by at least two reviewers and a third if necessary. All reviewers will be at PhD level or above and familiar with the phenomena under investigation.
Screening will be conducted in two stages.
1. MM and a second reviewer will independently screen titles and / or abstracts retrieved using the electronic search strategy against the eligibility criteria.
2. MM will retrieve the full text for potentially eligible studies and transfer them to EndNote. Full text articles will be independently screened by MM and a second reviewer.
Results from each pair of screeners will be merged and this list will be used in a consensus meeting at each screening stage. Any disagreement over the eligibility of particular studies will be resolved through discussion with a third reviewer.
MM will conduct the supplementary search strategy in consultation with SF.
A standardised, pre-piloted form will be used to extract data from the included studies for study description, critical appraisal of quality and evidence synthesis.
Following Campbell et al. (2011), verbatim text relating to findings and concepts in primary studies will be extracted for coding and synthesis. Thomas and Harden (2008) highlighted the difficulty in identifying qualitative data particularly when analysis by authors of primary studies is thin. They thus extracted the entire ‘results’ sections (and ‘abstracts’, where relevant) of included papers verbatim for coding within NVivo. We will also extract the entire text of ‘results’ sections and additionally, any text relating to findings, themes or concepts from the ‘discussion’ or ‘conclusion’ sections.
MM and a second reviewer will independently extract data from 10% of included papers. The reviewers will then meet to discuss approaches taken and any discrepancies. MM will independently extract data from the remaining articles in consultation with SF.

Risk of bias (quality) assessment

The quality of included studies will be appraised using EPPI-Centre criteria in Rees et al. (2009). MM and a second reviewer will independently appraise 10% of studies in terms of methodological rigour, reliability, trustworthiness and usefulness using a five-point scale. The reviewers will then meet to discuss approaches taken and any discrepancies. MM will independently appraise the remaining articles in consultation with SF.
Primary studies that otherwise satisfy the eligibility criteria will not be excluded on the basis of quality alone. Following Thomas and Harden (2008), a 'sensitivity analyses' will be undertaken to assess the possible impact of study quality on the review's findings by examining the relative contribution of each included study on the final themes.

Strategy for data synthesis

The review is interpretive or configurative as opposed to aggregative and aims to advance conceptual understanding or theory. The process of data synthesis will follow the thematic synthesis method outlined by Thomas & Harden (2008). The three stages of thematic synthesis will be undertaken within QSR’s NVivo, as follows:
1. Translating concepts in primary studies into one another by coding text ‘line-by-line’ and axially coding according to meaning and content to inductively develop initial descriptive codes. The coding process will be piloted as follows: a portion of the studies will be coded independently by MM and a second reviewer. The reviewers will meet to discuss the approaches taken and differences arising. A summary will be presented to the full review team for feedback. MM will continue the coding process in consultation with SF and in discussion with the full team.
2. Looking for similarities and differences among the descriptive codes in order to group them and organise into hierarchical structures, i.e. structuring the descriptive codes into ‘descriptive themes’. MM and a

second reviewer will conduct this level of analysis independently before meeting to discuss the approaches taken and differences arising. Once a final schema of descriptive themes has been agreed, MM will summarise and describe the themes in narrative form with input and feedback from the other review authors.
3. Generating interpretive 'analytical themes' that bring fresh insights, conceptual understanding or explanatory theory to the phenomenon. The descriptive themes that emerged from inductive analysis of primary study findings will be 'interrogated' in order to answer our synthesis review questions. As described by Thomas and Harden (2008), MM will use the emic perspectives captured by the descriptive themes to infer answers to the research questions and consider implications for interventions. MM will develop analytical themes that are 'sufficiently abstract to describe and / or explain all of our initial descriptive themes, our inferred barriers and facilitators and implications for intervention development'. MM will present the analysis to the other review authors for discussion, comment and revision.

Analysis of subgroups or subsets

Comparative analysis will be undertaken in order to explore possible patterns of association with participant characteristics such as age, sex, type and severity of aphasia, time post onset, living situation, study country and health system in terms of service availability, costs etc. (Bazeley, 2009). As this is a qualitative synthesis, it is not possible to specific all subgroup analysis we may conduct in advance.

Contact details for further information

Molly Manning
molly.manning@ul.ie

Organisational affiliation of the review

Department of Clinical Therapies, University of Limerick, Castletroy, County Limerick, Ireland

Review team members and their organisational affiliations

Ms Molly Manning. Department of Clinical Therapies, University of Limerick
Professor Anne Hickey. Department of Psychology, Royal College of Surgeons in Ireland
Professor Anne MacFarlane. Graduate Entry Medical School, University of Limerick
Professor Sue Franklin. Department of Clinical Therapies, University of Limerick

Anticipated or actual start date

01 October 2016

Anticipated completion date

31 July 2017

Funding sources/sponsors

This review is funded by the Health Research Board SPHeRE/2013/1.

Conflicts of interest

MM is a PhD scholar on a national structured PhD programme (SPHeRE) funded by the Health Research Board.
No other conflicts of interest are known.

Language

English

Country

Ireland

Stage of review

Review_Ongoing

Subject index terms status

Subject indexing assigned by CRD

Subject index terms

Activities of Daily Living; Aphasia; Humans; Physical and Rehabilitation Medicine; Neurological Rehabilitation; Patient Satisfaction; Stroke; Stroke Rehabilitation; Treatment Outcome

Date of registration in PROSPERO

27 January 2017

Date of publication of this version

27 January 2017

Details of any existing review of the same topic by the same authors

Stage of review at time of this submission

Stage	Started	Completed
Preliminary searches	Yes	Yes
Piloting of the study selection process	Yes	No
Formal screening of search results against eligibility criteria	No	No
Data extraction	No	No
Risk of bias (quality) assessment	No	No
Data analysis	No	No

Versions

27 January 2017

PROSPERO
This information has been provided by the named contact for this review. CRD has accepted this information in good faith and registered the review in PROSPERO. CRD bears no responsibility or liability for the content of this registration record, any associated files or external websites.
