# Supplementary material for: Perspectives of people with aphasia post-stroke towards personal recovery and living successfully: A systematic review and thematic synthesis
Source: PLoS One. 2019 Mar 22;14(3):e0214200. doi: 10.1371/journal.pone.0214200 (PMC6430359; doi:10.1371/journal.pone.0214200)
Supplement: S2 Text — (PDF) [file pone.0214200.s002.pdf]

S5 Study eligibility criteria

Inclusion criteria

Sample
- Adults aged 18+ years who have a diagnosis of aphasia as a result of stroke.
- No restrictions will be applied based on aphasia type or severity or any other demographic variable.

Research type, Design & Evaluation
- Studies in any language, from any publication date and any geographical location.
- Studies that seek to understand the perspectives, experiences, preferences and opinions of adults with aphasia post-stroke towards recovery and living successfully with aphasia, through direct contact with participants (interviews and focus group discussions) and qualitative data analysis. This includes:
    - Primary qualitative studies.
    - Studies that employ secondary analysis of qualitative data.
    - A qualitative study as part of a mixed methods study.

Phenomenon of interest
Experiences, needs, perceptions of adults with aphasia post-stroke towards:
- Personal recovery
- Living successfully with aphasia
- The lived experience of aphasia
- Rehabilitation, therapy, services
- Goals
- Friendship and relationships
- Community reintegration, participation, social participation
- Returning to work, volunteering
- Autonomy, Independence
- Renegotiating a new identity
- Coping, grief, loss, hope, other emotions
- Everyday occupations

S5 Study eligibility criteria

Exclusion criteria

HIERARCHY FOR RECORDING DECISIONS TO EXCLUDE CITATIONS: Sample – Research type – Evaluation – Phenomenon of Interest – Design.

<u>Sample</u>

- Children (<18 years).
- No diagnosis of stroke.
- Aphasia as a result of aetiologies other than stroke (e.g. TBI), unless results from people with aphasia are explicitly separate from other participants.
- Mixed groups of participants e.g. people with aphasia and their significant others or health care professionals, unless results from people with aphasia are explicitly separate from other participants.

<u>Research type</u>

- Any quantitative study, (RCT, non RCT, observational, cohort, case control).
- Any study where qualitative data is not analysed, i.e. uninterpreted data.
- Studies that do not state or describe a qualitative method of data analysis.
- Non-empirical articles including opinion pieces, commentaries, theoretical articles.
- Personal accounts that do not employ qualitative methods of data collection and analysis.
- Any review (systematic, narrative, qualitative).
- Treatment guidelines documents.
- Grey literature / not published in a peer reviewed journal.
- Dissertations/theses/conference proceedings/published abstracts – however attempts will be made to source full-length articles if abstracts are otherwise eligible.

<u>Evaluation</u>

- Any perspectives other than people with aphasia post-stroke.

<u>Phenomenon of interest</u>

- Any data not obtained through interview / focus group discussion methods.
- Papers describing experiences, perspectives in an experimental situation rather than ‘typical’, e.g. developing / evaluating a specific tool or intervention.
- Papers with a **specific** focus on communication, social communication or information needs.

<u>Design</u>

- Qualitative studies using questionnaires or other methods that do not involve interviews or focus group discussions with participants.
