# Supplementary material for: Perspectives of people with aphasia post-stroke towards personal recovery and living successfully: A systematic review and thematic synthesis
Source: PLoS One. 2019 Mar 22;14(3):e0214200. doi: 10.1371/journal.pone.0214200 (PMC6430359; doi:10.1371/journal.pone.0214200)
Supplement: S3 Text — (PDF) [file pone.0214200.s003.pdf]

S10 Descriptive themes

We created 85 descriptive codes and 16 descriptive themes, organised into 3 groupings (Figure 2). 'Personal acceptance and integration of aphasia' concerns the individual process of accepting and integrating aphasia and its impacts into one's life and self-identity. 'Social support and participation' concerns individual, attitudinal, environmental and structural characteristics that impact positively and negatively on social and community participation. 'Responsive, relevant and collaborative support services' concerns individual and service characteristics that impact on ability to perform self-care and to collaborate on, manage and direct one's treatment and recovery.

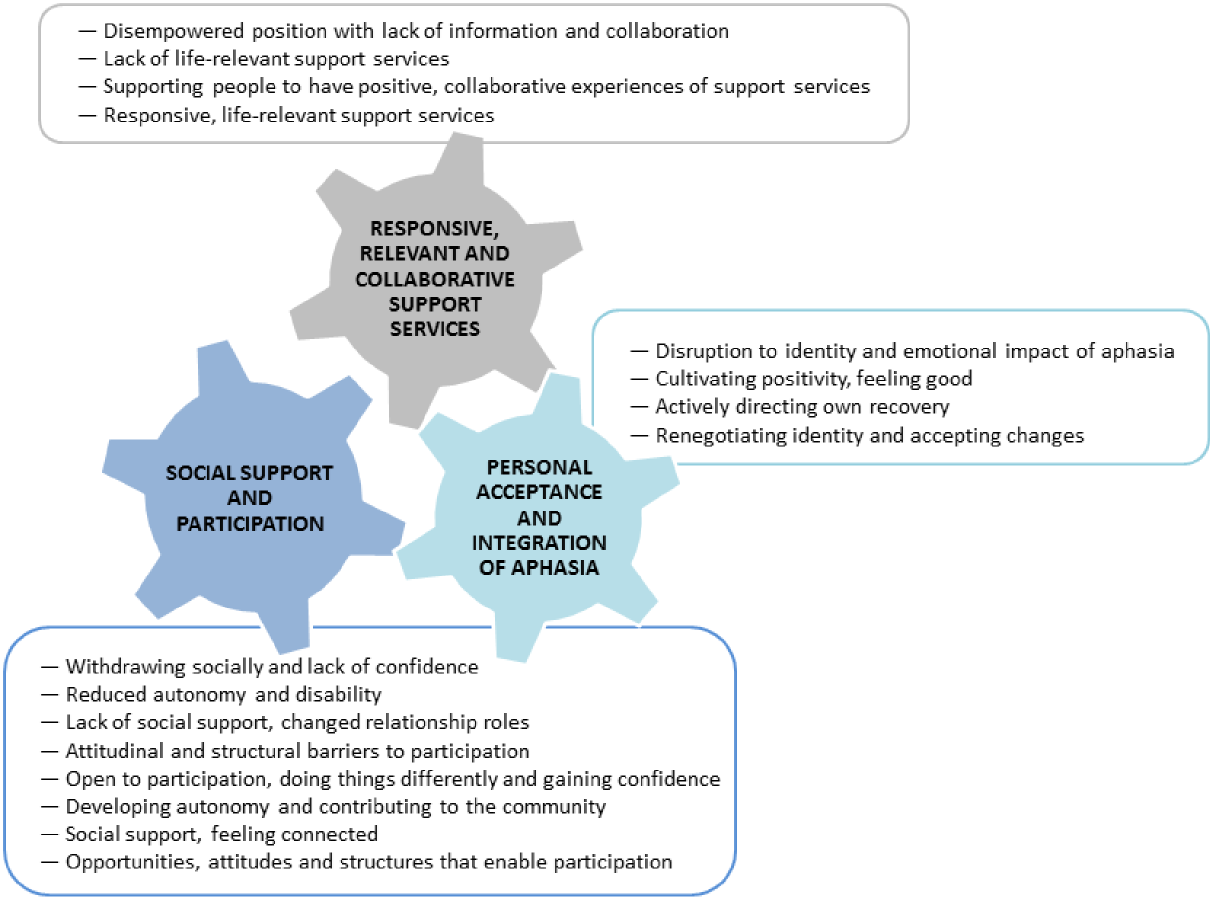

Fig. Descriptive themes
