# Supplementary material for: Perspectives of people with aphasia post-stroke towards personal recovery and living successfully: A systematic review and thematic synthesis
Source: PLoS One. 2019 Mar 22;14(3):e0214200. doi: 10.1371/journal.pone.0214200 (PMC6430359; doi:10.1371/journal.pone.0214200)
Supplement: S4 Text — (PDF) [file pone.0214200.s004.pdf]

S11 Barrier and enabler statements

BARRIERS

- The impacts of aphasia and stroke on psychosocial wellbeing, participation and autonomy are diversely and dynamically experienced in the long-term.
- Participation and autonomy are reduced due to aphasia and other impacts of stroke including other stroke-related disabilities (physical, cognitive and psychological), concomitant health conditions, reduced ability to get around independently, not living in one's own home and reduced income and ability to manage finances.
- Attitudinal and structural barriers to social and community participation include discrimination and restricted access to the work environment, public and commercial services and conversations.
- Restricted access to commercial and public facilities including legal, financial, banking, insurance and consumer services reduces participation and autonomy.
- Disabling attitudes and discrimination from others is linked with ignorance about aphasia.
- The invisible nature of aphasia may make it harder to explain and for others to understand.
- Returning to work or voluntary activities is a negatively experienced when there is a lack of appropriate support or mismatch of duties and skillsets.
- It may not be feasible to change all disabling aspects of contemporary lifestyle, for instance particular job demands.
- People experience negative changes to relationships and relationship roles including overprotection, a lack of tolerance and understanding, loss of friendships, reduced opportunity for social contact and isolation, which may arise through ignorance of aphasia and a lack of support and understanding from close others.
- Reduced ability to relate to others to a satisfying degree and a lack of confidence mean that people may withdraw from social interactions, avoid speaking in conversations and lack readiness for participation.
- Practical and emotional social support from friends and family are important for developing autonomy and participation, but increased dependence and loss of autonomy may be experienced as disempowering with a perceived loss of reciprocity in relationships.
- There is perceived tension between struggling to do things for oneself and accepting support in order to direct energy towards other activities.
- There is a lack of access to life-relevant supports that help people to come to terms with their aphasia and to direct their own recovery.
- People experience financial, geographical, informational, attitudinal barriers to accessing services.

S11 Barrier and enabler statements

- Navigating and interacting with health services is disempowering due to a lack of information, a lack of collaboration and a lack of knowledge and training about aphasia on the part of health professionals.
- Aphasia increases vulnerability and potential for adverse events in hospital and rehabilitation settings.
- A lack of information about aphasia and service delivery acts as a barrier to making sense of aphasia and restricts people with aphasia from directing their own recovery and self-advocating for appropriate support.
- Negative prognostic information disrupts hope, positivity and engagement with rehabilitation.
- There is a lack of support in the long-term for relatively younger people with aphasia, for whom there may be specific impacts in terms of parenting roles and reduced income.
- Aphasia occurs in the context of a wider social support network that is relied upon for support, acceptance and encouragement but is not supported.
- Irrelevant, theoretical and patronising language therapy is linked with a lack of collaboration and may lead to patient-led discharge and disinterest in further support.

ENABLERS

- Coming to terms with and accepting aphasia, renegotiating identity, cultivating positivity and actively directing recovery and self-growth require long-term perseverance and effort.
- Being proactive about participation socialising, trying new activities and being open to adapting and doing things differently facilitates participation, success and wellbeing.
- Being open about aphasia, using communication strategies and being assertive in the face of disabling attitudes helps to raise awareness and to maximise participation and autonomy.
- Social and community participation are supported by increased public awareness of aphasia, supportive conversation partners and via strategies, policies and procedures that support people in the workplace, organisations or public services.
- Participation is enabled by opportunities to socialise with others including attending groups and opportunities to meet other PWA, often through therapy contexts.
- People with aphasia want to be recognised as useful and contributing to society and / or their community through actions such as returning to work, volunteering and helping others.
- They recognise their potential for supporting others going through similar experiences.

S11 Barrier and enabler statements

- Participation provides opportunities to learn new capacity and new skills and to explore retained skills which help to develop confidence.
- Engaging in activities and doing things for oneself supports sense of autonomy and control
- Increased confidence through participation supports subsequent participation and independent engagement in higher levels of activity.
- Participation and autonomy are supported by ability to get around independently, good health, fitness and mobility.
- Meaningful relationships, maintaining relationships roles such as parenting, acceptance and understanding, and spending time and feeling connected with family and friends are important for a sense of living successfully.
- Meeting others with aphasia and stroke provides a valued opportunity for new friendships, support, information and making sense of aphasia, a sense of belonging and mutual understanding.
- Life-relevant language therapy is valued in terms of improving communicative function and participation and as a source of information, ideas and resources for directing one's own recovery.
- Access to services must be flexible and individualised to support people to terms with the dynamic and evolving impacts of aphasia, to target life-relevant goals and to self-manage recovery and self-growth in the long-term.
- PWA value positive therapeutic interactions that cultivate and foster hope and enhance the relevance of therapeutic goals and activities.
- People value HCPs who are knowledgeable about aphasia and how to support their communication needs.
- Support with communication and memory from family members is often required to compensate for the shortcomings of medical professionals and the disabling nature of interactions with healthcare professionals.
- People need support for coping with negative emotions and coming to terms with multiple losses.
- Access to information about aphasia and services supports people to make sense of and to come to terms with aphasia, to direct their own recovery and to manage their care.
- People are differentially able / interested in collaboration depending on their stage of recovery.
- Involving PWA in service planning enhances the relevance of activities and the status of an organisation.

S11 Barrier and enabler statements

- Formal and informal supports accessed in the community and which support recovery, participation and autonomy include librarian-led reading support, nursing care and home help.
