# Supplementary material for: Perspectives of people with aphasia post-stroke towards personal recovery and living successfully: A systematic review and thematic synthesis
Source: PLoS One. 2019 Mar 22;14(3):e0214200. doi: 10.1371/journal.pone.0214200 (PMC6430359; doi:10.1371/journal.pone.0214200)
Supplement: S1 Checklist — (PDF) [file pone.0214200.s005.pdf]

S2 ENTREQ checklist

Enhancing transparency in reporting the synthesis of qualitative research: the ENTREQ statement

No	Item	Guide and description	Page
**1**	Aim	State the research question the synthesis addresses.	8-9
**2**	Synthesis methodology	Identify the synthesis methodology or theoretical framework which underpins the synthesis, and describe the rationale for choice of methodology *(e.g. meta-ethnography, thematic synthesis, critical interpretive synthesis, grounded theory synthesis, realist synthesis, meta-aggregation, meta-study, framework synthesis).*	10
**3**	Approach to searching	Indicate whether the search was pre-planned (*comprehensive search strategies to seek all available studies)* or iterative (*to seek all available concepts until they theoretical saturation is achieved)*.	10
**4**	Inclusion criteria	Specify the inclusion/exclusion criteria *(e.g. in terms of population, language, year limits, type of publication, study type).*	10-11, S5
**5**	Data sources	Describe the information sources used (e.g. *electronic databases (MEDLINE, EMBASE, CINAHL, psycINFO, Econlit), grey literature databases (digital thesis, policy reports), relevant organisational websites, experts, information specialists, generic web searches (Google Scholar) hand searching, reference lists)* and when the searches conducted; provide the rationale for using the data sources.	10, S4
**6**	Electronic Search strategy	Describe the literature search *(e.g. provide electronic search strategies with population terms, clinical or health topic terms, experiential or social phenomena related terms, filters for qualitative research, and search limits)*.	S4
**7**	Study screening methods	Describe the process of study screening and sifting *(e.g. title, abstract and full text review, number of independent reviewers who screened studies).*	10-11, S5
**8**	Study characteristics	Present the characteristics of the included studies *(e.g. year of publication, country, population, number of participants, data collection, methodology, analysis, research questions).*	13, S8
**9**	Study selection results	Identify the number of studies screened and provide reasons for study exclusion *(e,g, for comprehensive searching, provide numbers of studies screened and reasons for exclusion indicated in a figure/flowchart; for iterative searching describe reasons for study exclusion and inclusion based on modifications t the research question and/or contribution to theory development).*	12-13, Fig 1
**10**	Rationale for appraisal	Describe the rationale and approach used to appraise the included studies or selected findings *(e.g. assessment of conduct (validity and robustness), assessment of reporting (transparency), assessment of content and utility of the findings).*	11, S6
**11**	Appraisal items	State the tools, frameworks and criteria used to appraise the studies or selected findings *(e.g. Existing tools: CASP, QARI, COREQ, Mays and Pope* [25]*; reviewer developed tools; describe the domains assessed: research team, study design, data analysis and interpretations, reporting).*	11, S6
**12**	Appraisal process	Indicate whether the appraisal was conducted independently by more than one reviewer and if consensus was required.	11
**13**	Appraisal results	Present results of the quality assessment and indicate which articles, if any, were weighted/excluded based on the assessment and give the rationale.	13-14, S9

S2 ENTREQ checklist

No	Item	Guide and description	Page
**14**	Data extraction	Indicate which sections of the primary studies were analysed and how were the data extracted from the primary studies? *(e.g. all text under the headings "results /conclusions" were extracted electronically and entered into a computer software).*	11
**15**	Software	State the computer software used, if any.	11
**16**	Number of reviewers	Identify who was involved in coding and analysis.	12
**17**	Coding	Describe the process for coding of data *(e.g. line by line coding to search for concepts).*	12
**18**	Study comparison	Describe how were comparisons made within and across studies *(e.g. subsequent studies were coded into pre-existing concepts, and new concepts were created when deemed necessary).*	12
**19**	Derivation of themes	Explain whether the process of deriving the themes or constructs was inductive or deductive.	12
**20**	Quotations	Provide quotations from the primary studies to illustrate themes/constructs, and identify whether the quotations were participant quotations of the author's interpretation.	14-23
**21**	Synthesis output	Present rich, compelling and useful results that go beyond a summary of the primary studies (e.g. *new interpretation, models of evidence, conceptual models, analytical framework, development of a new theory or construct).*	14-23
