# Supplementary material for: Perspectives of people with aphasia post-stroke towards personal recovery and living successfully: A systematic review and thematic synthesis
Source: PLoS One. 2019 Mar 22;14(3):e0214200. doi: 10.1371/journal.pone.0214200 (PMC6430359; doi:10.1371/journal.pone.0214200)
Supplement: S1 Table — (PDF) [file pone.0214200.s007.pdf]

S4 Search strategy

We designed and optimised a single line search syntax (Sample AND (Design OR Evaluation OR Research type) AND Phenomenon of Interest) in EMBASE using free text terms and Emtree headings and synonyms before translating for Medline (Ovid), PubMed, Web-of-Science, Scopus, CINAHL and PsycINFO (EBSCO) and MeSH and CINAHL headings.

Translation of thesaurus and free text terms:

	Emtree	MeSH	CINAHL headings	PsycINFO headings	Free text terms
S	“aphasia”/	aphasia/	(MH "Aphasia")	(DE "Aphasia")	aphasia, aphasic, dysphasic, dysphasia
D OR E OR R	‘qualitative research’/exp OR ‘qualitative analysis’/de OR ‘ethnography’/de OR ‘action research’/de OR ‘participatory research’/de OR ‘triangulation’/de OR ‘grounded theory’/de OR ‘exploratory research’/de OR ‘interview’/exp OR ‘naturalistic inquiry’/de OR ‘phenomenology’/de OR ‘patient attitude’/de OR ‘patient preference’/de	"qualitative research"/ OR "community-based participatory research"/ OR "focus groups"/ OR "hermeneutics"/ OR "health services research"/ OR "grounded theory"/ OR "patient preference"/	MH "Qualitative Studies+" OR MH "Qualitative Validity+" OR MH "Phenomenology" OR MH "Health Services Research+" OR MH "Grounded Theory" OR MH "Interviews+" OR MH "Focus Groups" OR MH "Observational Methods+" OR MH "Patient Attitudes" OR MH "Attitude to Illness"	(DE "Qualitative Research" OR DE "Interviews" OR DE "Grounded Theory" OR DE "Observation Methods" OR DE "Phenomenology" OR DE "Client Attitudes" OR DE "Hermeneutics" OR DE "Constructivism")	qualitative, “action research”, “grounded theory”, exploratory, “naturalistic inquiry”. phenomenolog*, triangulat*. hermeneutic*, interview*, “focus group*”, participat*, perspective*, experience*, perception*, attitude*, opinion*, preference*, perceive, construe, hope*, wish*, view*, ethnograph*, metaethnography, meta-ethnography, think, thoughts, feel*, constructivis*, interpretiv*, (analy* ADJ4 inductive*), (analy* ADJ4 thematic*)

S4 Search strategy

PI	‘return to work’/de OR ‘lifestyle and related phenomena’/exp OR ‘education’/exp OR ‘lifelong learning’/de OR ‘access to information’/de ‘social acceptance’/exp OR ‘self care’/de OR ‘self help’/de OR ‘quality of life’/de OR ‘identity’/de OR ‘social participation’/de OR ‘human activities’/exp OR ‘emotion’/exp OR ‘wellbeing’/de OR ‘psychological wellbeing’/de OR ‘self concept’/exp OR ‘human relation’/de OR ‘friendship’/de OR ‘intimacy’/de OR ‘social network’/de OR ‘lifestyle and related phenomena’/exp OR ‘occupation’/exp OR ‘social environment’/exp OR ‘personal autonomy’/de OR ‘self esteem’/de OR ‘patient autonomy’/de OR ‘social support’/de	exp "work"/ OR exp "leisure activities"/ OR exp "social environment"/ OR exp "interpersonal relations"/ OR "social participation"/ OR "social support"/ OR "patient participation"/ OR "psychological support systems"/ OR "self management"/ OR "friends"/ OR "self care"/ OR "health care quality, access and evaluation"/ OR "quality of life"/ OR "self concept"/ OR "personal autonomy"/ OR exp "interpersonal relations"/ OR "return to work"/ OR "employment"/ OR "education"/ OR "lifelong learning"/	(MH "Quality of Life") OR (MH “Personal Growth”) OR (MH “Life Purpose”) OR (MH "Social Participation") OR (MH "Support, Psychosocial+") OR (MH "Access to Information") OR (MH "Autonomy+") OR (MH "Self Care") OR (MH "Self Concept") OR (MH "Social Identity") OR (MH "Emotions+") OR (MH "Interpersonal Relations+") OR (MH "Life Style+") OR (MH "Employment+") OR (MH "Leisure Activities+")	(DE "Quality of Life" OR DE "Recovery (Disorders)" OR (DE "Community Involvement" OR DE "Participation" OR DE "Social Support" OR DE "Emotions" OR DE "Autonomy" OR DE "Independence (Personality)" OR DE "Empowerment" OR DE "Self-Care Skills" OR DE "Self-Concept" OR DE "Leisure Time" OR DE "Meaningfulness" OR DE "Friendship" OR DE "Intimacy")	“return to work”, employment, education, “lifelong learning”, (access ADJ3 information), acceptance, ((live OR living) ADJ3 successful*), (life ADJ3 quality), HRQL, self-<br>care, self-help, “sick role”, identity, impact*, adapt*, cope*, coping, self-efficacy self-image, self-concept, self-management, wellbeing, well-being, wellness, participat*, social*, friend*, relationship*, intimacy, emotion*, lifestyle, “personal experience”, “satisfaction”, journey, inclusion, occupation, income, drive, driving, “going out”, ((life OR liv* OR experience) ADJ3 stroke), ((life OR liv* OR experience) ADJ3 aphasia), (role ADJ3 change), “everyday life”, recovery, meaningful*

S4 Search strategy

Single string search syntax by database S AND (D OR E OR R) AND PI

**EMBASE** (ELSEVIER PLATFORM)	(('aphasia'/de OR (aphasia OR dysphasia OR aphasic OR dysphasic):ab,ti) AND (‘qualitative research’/exp OR ‘qualitative analysis’/de OR ‘ethnography’/de OR ‘action research’/de OR ‘participatory research’/de OR ‘triangulation’/de OR ‘grounded theory’/de OR ‘exploratory research’/de OR ‘interview’/exp OR ‘naturalistic inquiry’/de OR ‘phenomenology’/de OR ‘patient attitude’/de OR ‘patient preference’/de OR (qualitative OR ‘action research’ OR ‘grounded theory’ OR exploratory OR ‘naturalistic inquiry’ OR ‘focus group’ OR ‘focus groups’ OR ethnograph* OR metaethnography OR meta-ethnography OR phenomenology* OR triangulat* OR hermeneutic* OR interview* OR participat* OR perspective* OR experience* OR perception* OR perceive OR construe OR attitude* OR opinion* OR preference* OR hope* OR wish* OR view* OR think OR thoughts OR feel* or constructivis* OR interpretiv* OR (analy* NEAR/4 inductive*) OR (analy* NEAR/4 thematic*)):ab,ti) AND (‘return to work’/de OR ‘lifestyle and related phenomena’/exp OR ‘education’/exp OR ‘lifelong learning’/de OR ‘access to information’/de ‘social acceptance’/exp OR ‘self care’/de OR ‘self help’/de OR ‘quality of life’/de OR ‘identity’/de OR ‘social participation’/de OR ‘human activities’/exp OR ‘emotion’/exp OR ‘wellbeing’/de OR ‘psychological wellbeing’/de OR ‘self concept’/exp OR ‘human relation’/de OR ‘friendship’/de OR ‘intimacy’/de OR ‘social network’/de OR ‘lifestyle and related phenomena’/exp OR ‘occupation’/exp OR ‘social environment’/exp OR ‘personal autonomy’/de OR ‘self esteem’/de OR ‘patient autonomy’/de OR ‘social support’/de OR (‘return to work’ OR employment OR education OR ‘lifelong learning’ OR (access NEAR/3 information) OR acceptance OR ((live OR living) NEAR/3 successful*) OR (life NEAR/3 quality) OR HRQL OR self-care OR self-help OR ‘sick role’ OR identity OR impact* OR adapt* OR cope* OR coping OR self-efficacy OR self-image OR self-concept OR self-management OR wellbeing OR well-being OR wellness OR participat* OR social* OR friend* OR relationship* OR intimacy OR emotion* OR lifestyle OR ‘personal experience’ OR satisfaction OR journey OR inclusion OR occupation OR income OR drive OR driving OR ‘going out’ OR ((life OR liv* OR experience) NEAR/3 stroke) OR ((life OR liv* OR experience) NEAR/3 aphasia) OR (role NEAR/3 change) OR ‘everyday life’ OR independence OR autonomy OR self-esteem OR meaningful* OR recovery):ab,ti))
**WEB OF SCIENCE**	TS=((aphasia OR dysphasia OR aphasic OR dysphasic) AND (qualitative OR "action research" OR "grounded theory" OR exploratory OR "naturalistic inquiry" OR "focus group" OR "focus groups" OR ethnograph* OR metaethnography OR meta-ethnography OR phenomenology* OR triangulat* OR hermeneutic* OR interview* OR participat* OR perspective* OR experience* OR perception* OR perceive OR construe OR attitude* OR opinion* OR preference* OR hope* OR wish* OR view* OR think OR thoughts OR feel* or constructivis* OR interpretiv* OR (analy* NEAR/4 inductive*) OR (analy* NEAR/4 thematic*)) AND ("return to work" OR employment OR education OR "lifelong learning" OR (access NEAR/2 information) OR acceptance OR ((live OR living) NEAR/2 successful*) OR (life NEAR/2 quality) OR HRQL OR self-care OR self-help OR "sick role" OR identity OR impact* OR adapt* OR cope* OR coping OR self-efficacy OR self-image OR self-concept OR self-management OR wellbeing OR well-being OR wellness OR participat* OR social* OR friend* OR relationship* OR intimacy OR emotion* OR lifestyle OR "personal experience" OR satisfaction OR journey OR inclusion OR occupation OR income OR drive OR driving OR "going out" OR ((life OR liv* OR experience) NEAR/2 stroke) OR ((life OR liv* OR experience) NEAR/2 aphasia) OR (role NEAR/2 change) OR "everyday life" OR independence OR autonomy OR self-esteem OR meaningful* OR recovery))
**MEDLINE (Ovid)**	(("aphasia"/ OR (aphasia OR dysphasia OR aphasic OR dysphasic).ab,ti.) AND ("qualitative research"/ OR "community-based participatory research"/ OR "focus groups"/ OR "hermeneutics"/ OR "health services research"/ OR "grounded theory"/ OR "patient preference"/ OR (qualitative OR "action research" OR "grounded theory" OR exploratory OR "naturalistic inquiry" OR "focus group" OR "focus groups" OR ethnograph* OR metaethnography OR meta-

S4 Search strategy

	ethnography OR phenomenology* OR triangulat* OR hermeneutic* OR interview* OR participat* OR perspective* OR experience* OR perception* OR perceive OR construe OR attitude* OR opinion* OR preference* OR hope* OR wish* OR view* OR think OR thoughts OR feel* or constructivis* OR interpretiv* OR (analy* ADJ4 inductive*) OR (analy* ADJ4 thematic*)).ab,ti.) AND (exp "work"/ OR exp "leisure activities"/ OR exp "social environment"/ OR exp "interpersonal relations"/ OR "social participation"/ OR "social support"/ OR "patient participation"/ OR "psychological support systems"/ OR "self management"/ OR "friends"/ OR "self care"/ OR "health care quality, access and evaluation"/ OR "quality of life"/ OR "self concept"/ OR "personal autonomy"/ OR exp "interpersonal relations"/ OR "return to work"/ OR "employment"/ OR "education"/ OR "lifelong learning"/ OR (" return to work" OR " employment" OR " education" OR " lifelong learning" OR (access ADJ3 information) OR acceptance OR ((live OR living) ADJ3 successful*) OR (life ADJ3 quality) OR HRQL OR self-care OR self-help OR "sick role" OR identity OR impact* OR adapt* OR cope* OR coping OR self-efficacy OR self-image OR self-concept OR self-management OR wellbeing OR well-being OR wellness OR participat* OR social* OR friend* OR relationship* OR intimacy OR emotion* OR lifestyle OR "personal experience" OR satisfaction OR journey OR inclusion OR occupation OR income OR drive OR driving OR "going out" OR ((life OR liv* OR experience) ADJ3 stroke) OR ((life OR liv* OR experience) ADJ3 aphasia) OR (role ADJ3 change) OR "everyday life" OR independence OR autonomy OR self-esteem OR meaningful* OR recovery).ab,ti.))
**PUBMED** (excluding MEDLINE content)	("aphasia"[mh] OR aphasia[tiab] OR dysphasia[tiab] OR aphasic[tiab] OR dysphasic[tiab]) AND ("qualitative research"[mh] OR "community-based participatory research"[mh] OR "focus groups"[mh] OR "hermeneutics"[mh] OR "health services research"[mh] OR "grounded theory"[mh] OR "patient preference"[mh] OR qualitative[tiab] OR "action research"[tiab] OR "grounded theory"[tiab] OR exploratory[tiab] OR "naturalistic inquiry"[tiab] OR "focus group"[tiab] OR "focus groups"[tiab] OR ethnograph*[tiab] OR metaethnography[tiab] OR meta-ethnography[tiab] OR phenomenolog*[tiab] OR triangulat*[tiab] OR hermeneutic*[tiab] OR interview*[tiab] OR participat*[tiab] OR perspective*[tiab] OR experience*[tiab] OR perception*[tiab] OR perceive[tiab] OR construe[tiab] OR attitude*[tiab] OR opinion*[tiab] OR preference*[tiab] OR hope*[tiab] OR wish*[tiab] OR view*[tiab] OR think[tiab] OR thoughts[tiab] OR feel*[tiab] or constructivis*[tiab] OR interpretiv*[tiab] OR "inductive analysis"[tiab] OR "thematic analysis"[tiab]) AND ("work"[mh] OR "leisure activities"[mh] OR "social environment"[mh] OR "interpersonal relations"[mh] OR "social participation"[mh] OR "social support"[mh] OR "patient participation"[mh] OR "psychological support systems"[mh] OR "self management"[mh] OR "friends"[mh] OR "self care"[mh] OR "health care quality, access and evaluation"[mh] OR "quality of life"[mh] OR "self concept"[mh] OR "personal autonomy"[mh] OR "interpersonal relations"[mh] OR "return to work"[mh] OR "employment"[mh] OR "education"[mh] OR "lifelong learning"[mh] OR "return to work"[tiab] OR employment[tiab] OR education[tiab] OR "lifelong learning"[tiab] OR "access to information"[tiab] OR "information access"[tiab] OR acceptance[tiab] OR "live successfully"[tiab] OR "living successfully"[tiab] OR "successful living"[tiab] OR "life quality"[tiab] OR "quality of life"[tiab] OR HRQL[tiab] OR self-care[tiab] OR self-help[tiab] OR "sick role"[tiab] OR identity[tiab] OR impact*[tiab] OR adapt*[tiab] OR cope*[tiab] OR coping[tiab] OR self-efficacy[tiab] OR self-image[tiab] OR self-concept[tiab] OR self-management[tiab] OR wellbeing[tiab] OR well-being[tiab] OR wellness[tiab] OR participat*[tiab] OR social*[tiab] OR friend*[tiab] OR relationship*[tiab] OR intimacy[tiab] OR emotion*[tiab] OR lifestyle[tiab] OR "personal experience"[tiab] OR satisfaction[tiab] OR journey[tiab] OR inclusion[tiab] OR occupation[tiab] OR income[tiab] OR drive[tiab] OR driving[tiab] OR "going out"[tiab] OR "lived experience"[tiab] OR "role"[tiab] OR

S4 Search strategy

	"everyday life"[tiab] OR independence[tiab] OR autonomy[tiab] OR self-esteem[tiab] OR meaningful*[tiab] OR recovery[tiab]) AND publisher[sb]
**SCOPUS**	TITLE-ABS-KEY((aphasia OR dysphasia OR aphasic OR dysphasic) AND (qualitative OR “action research” OR “grounded theory” OR exploratory OR “naturalistic inquiry” OR “focus group” OR “focus groups” OR ethnograph* OR metaethnography OR meta-ethnography OR phenomenology* OR triangulat* OR hermeneutic* OR interview* OR participat* OR perspective* OR experience* OR perception* OR perceive OR construe OR attitude* OR opinion* OR preference* OR hope* OR wish* OR view* OR think OR thoughts OR feel* or constructivis* OR interpretiv* OR (analy* W/4 inductive*) OR (analy* W/4 thematic*)) AND (“return to work” OR employment OR education OR “lifelong learning” OR (access W/2 information) OR acceptance OR ((live OR living) W/2 successful*) OR (life W/2 quality) OR HRQL OR self-care OR self-help OR “sick role” OR identity OR impact* OR adapt* OR cope* OR coping OR self-efficacy OR self-image OR self-concept OR self-management OR wellbeing OR well-being OR wellness OR participat* OR social* OR friend* OR relationship* OR intimacy OR emotion* OR lifestyle OR “personal experience” OR satisfaction OR journey OR inclusion OR occupation OR income OR drive OR driving OR “going out” OR ((life OR liv* OR experience) W/2 stroke) OR ((life OR liv* OR experience) W/2 aphasia) OR (role W/2 change) OR “everyday life” OR independence OR autonomy OR self-esteem OR meaningful* OR recovery))
**CINAHL**	(((MH "aphasia+”) OR TI(aphasia OR dysphasia OR aphasic OR dysphasic) OR AB(aphasia OR dysphasia OR aphasic OR dysphasic)) AND ((MH "Qualitative Studies+") OR (MH "Qualitative Validity+") OR (MH "Phenomenology") OR (MH "Health Services Research+") OR (MH "Grounded Theory") OR (MH "Interviews+") OR (MH "Focus Groups") OR (MH "Observational Methods+") OR (MH "Patient Attitudes") OR (MH "Attitude to Illness") OR TI(qualitative OR "action research" OR "grounded theory" OR exploratory OR "naturalistic inquiry" OR "focus group" OR "focus groups" OR ethnograph* OR metaethnography OR meta-ethnography OR phenomenology* OR triangulat* OR hermeneutic* OR interview* OR participat* OR perspective* OR experience* OR perception* OR perceive OR construe OR attitude* OR opinion* OR preference* OR hope* OR wish* OR view* OR think OR thoughts OR feel* or constructivis* OR interpretiv* OR (analy* N4 inductive*) OR (analy* N4 thematic*)) OR AB(qualitative OR "action research" OR "grounded theory" OR exploratory OR "naturalistic inquiry" OR "focus group*" OR ethnograph* OR metaethnography OR meta-ethnography OR phenomenology* OR triangulat* OR hermeneutic* OR interview* OR participat* OR perspective* OR experience* OR perception* OR perceive OR construe OR attitude* OR opinion* OR preference* OR hope* OR wish* OR view* OR think OR thoughts OR feel* or constructivis* OR interpretiv* OR (analy* N4 inductive*) OR (analy* N4 thematic*))) AND (((MH "Quality of Life") OR (MH “Personal Growth”) OR (MH “Life Purpose”) OR (MH "Social Participation") OR (MH "Support, Psychosocial+") OR (MH "Access to Information") OR (MH "Autonomy+") OR (MH "Self Care") OR (MH "Self Concept") OR (MH "Social Identity") OR (MH "Emotions+") OR (MH "Interpersonal Relations+") OR (MH "Life Style+") OR (MH "Employment+") OR (MH "Leisure Activities+") OR TI(" return to work" OR employment OR education OR "lifelong learning" OR (access N2 information) OR acceptance OR ((live OR living) N2 successful*) OR (life N2 quality) OR HRQL OR self-care OR self-help OR "sick role" OR identity OR impact* OR adapt* OR cope* OR coping OR self-efficacy OR self-image OR self-concept OR self-management OR wellbeing OR well-being OR wellness OR participat* OR social* OR friend* OR relationship* OR intimacy OR emotion* OR lifestyle OR "personal experience" OR satisfaction OR journey OR inclusion OR occupation OR income OR drive OR driving OR "going out" OR ((life OR liv* OR experience) N2 stroke) OR ((life OR liv* OR experience) N2 aphasia) OR (role N2 change) OR "everyday life" OR independence OR autonomy OR self-esteem OR meaningful* OR recovery) OR AB (" return to work" OR employment OR education OR "lifelong learning" OR (access N2 information) OR acceptance OR ((live OR living) N2 successful*) OR (life N2 quality) OR HRQL OR self-care OR self-help OR "sick role" OR identity OR impact* OR adapt* OR cope* OR

S4 Search strategy

	coping OR self-efficacy OR self-image OR self-concept OR self-management OR wellbeing OR well-being OR wellness OR participat* OR social* OR friend* OR relationship* OR intimacy OR emotion* OR lifestyle OR "personal experience" OR satisfaction OR journey OR inclusion OR occupation OR income OR drive OR driving OR "going out" OR ((life OR liv* OR experience) N2 stroke) OR ((life OR liv* OR experience) N2 aphasia) OR (role N2 change) OR "everyday life" OR independence OR autonomy OR self-esteem OR meaningful* OR recovery))))
**PSYCINFO (EBSCO)**	(((DE "Aphasia") OR TI(aphasia OR dysphasia OR aphasic OR dysphasic) OR AB(aphasia OR dysphasia OR aphasic OR dysphasic)) AND ((DE "Qualitative Research" OR DE "Interviews" OR DE "Grounded Theory" OR DE "Observation Methods" OR DE "Phenomenology" OR DE "Client Attitudes" OR DE "Hermeneutics" OR DE "Constructivism") OR TI(qualitative OR "action research" OR "grounded theory" OR exploratory OR "naturalistic inquiry" OR "focus group" OR "focus groups" OR ethnograph* OR metaethnography OR meta-ethnography OR phenomenology* OR triangulat* OR hermeneutic* OR interview* OR participat* OR perspective* OR experience* OR perception* OR perceive OR construe OR attitude* OR opinion* OR preference* OR hope* OR wish* OR view* OR think OR thoughts OR feel* or constructivis* OR interpretiv* OR (analy* N4 inductive*) OR (analy* N4 thematic*)) OR AB(qualitative OR "action research" OR "grounded theory" OR exploratory OR "naturalistic inquiry" OR "focus group*" OR ethnograph* OR metaethnography OR meta-ethnography OR phenomenology* OR triangulat* OR hermeneutic* OR interview* OR participat* OR perspective* OR experience* OR perception* OR perceive OR construe OR attitude* OR opinion* OR preference* OR hope* OR wish* OR view* OR think OR thoughts OR feel* or constructivis* OR interpretiv* OR (analy* N4 inductive*) OR (analy* N4 thematic*))) AND ((DE "Quality of Life" OR DE "Recovery (Disorders)" OR (DE "Community Involvement" OR DE "Participation" OR DE "Social Support" OR DE "Emotions" OR DE "Autonomy" OR DE "Independence (Personality)" OR DE "Empowerment" OR DE "Self-Care Skills" OR DE "Self-Concept" OR DE "Leisure Time" OR DE "Meaningfulness" OR DE "Friendship" OR DE "Intimacy") OR TI("return to work" OR employment OR education OR "lifelong learning" OR (access N2 information) OR acceptance OR ((live OR living) N2 successful*) OR (life N2 quality) OR HRQL OR self-care OR self-help OR "sick role" OR identity OR impact* OR adapt* OR cope* OR coping OR self-efficacy OR self-image OR self-concept OR self-management OR wellbeing OR well-being OR wellness OR participat* OR social* OR friend* OR relationship* OR intimacy OR emotion* OR lifestyle OR "personal experience" OR satisfaction OR journey OR inclusion OR occupation OR income OR drive OR driving OR "going out" OR ((life OR liv* OR experience) N2 stroke) OR ((life OR liv* OR experience) N2 aphasia) OR (role N2 change) OR "everyday life" OR independence OR autonomy OR self-esteem OR meaningful* OR recovery) OR AB (" return to work" OR employment OR education OR "lifelong learning" OR (access N2 information) OR acceptance OR ((live OR living) N2 successful*) OR (life N2 quality) OR HRQL OR self-care OR self-help OR "sick role" OR identity OR impact* OR adapt* OR cope* OR coping OR self-efficacy OR self-image OR self-concept OR self-management OR wellbeing OR well-being OR wellness OR participat* OR social* OR friend* OR relationship* OR intimacy OR emotion* OR lifestyle OR "personal experience" OR satisfaction OR journey OR inclusion OR occupation OR income OR drive OR driving OR "going out" OR ((life OR liv* OR experience) N2 stroke) OR ((life OR liv* OR experience) N2 aphasia) OR (role N2 change) OR "everyday life" OR independence OR autonomy OR self-esteem OR meaningful* OR recovery))))
