# Supplementary material for: Perspectives of people with aphasia post-stroke towards personal recovery and living successfully: A systematic review and thematic synthesis
Source: PLoS One. 2019 Mar 22;14(3):e0214200. doi: 10.1371/journal.pone.0214200 (PMC6430359; doi:10.1371/journal.pone.0214200)
Supplement: S2 Table — (PDF) [file pone.0214200.s008.pdf]

S6 Critical appraisal instrument

Adapted from Rees R, Oliver K, Woodman J, Thomas J. Children's views about obesity, body size, shape and weight: a systematic review. London: EPPI Centre, Social Science Research Unit, Institute of Education, University of London; 2009.

1	**Were steps taken to increase rigour in the sampling?**	**Select one:**
	Consider whether:<br>- the sampling strategy was appropriate to the questions posed in the study (e.g. was the strategy well reasoned and justified?);<br>- attempts were made to obtain a diverse sample of the population in question<br>(think about who might have been excluded; who may have had a different perspective to offer);<br>- characteristics of the sample critical to the understanding of the study context and findings were presented (i.e. do we know who the participants were in terms of, for example, basic socio-demographics, characteristics relevant to the context of the study, etc.).	1.1 Yes, a fairly thorough attempt was made<br><br>1.2 Yes, several steps were taken<br><br>1.3 Yes, minimal few steps were taken<br><br>1.4 No, not at all/ Not stated/Can't tell
**2**	**Were steps taken to increase rigour in the data collected?**	**Select one:**
	Consider whether:<br>- data collection tools were piloted;<br>- data collection was comprehensive, flexible and/or sensitive enough to provide a complete and/or vivid and rich description of people's perspectives and experiences (e.g. did the researchers spend sufficient time at the site/with participants? Did they keep 'following up'? Was more than one method of data collection used?);<br>- steps were taken to ensure that all participants were able and willing to contribute (e.g. processes for consent, language barriers, power relations between researchers and people with aphasia and significant others, where relevant).	2.1 Yes, a fairly thorough attempt was made<br><br>2.2 Yes, several steps were taken<br><br>2.3 Yes, minimal few steps were taken<br><br>2.4 No, not at all/ Not stated/Can't tell
**3**	**Were steps taken to increase rigour in the data analysis?**	**Select one:**
	Consider whether:<br>- data analysis methods were systematic (e.g. was a method described/can a method be discerned?);<br>- diversity in perspective was explored;<br>- the analysis was balanced in the extent to which it was guided by preconceptions or by the data;<br>- the analysis sought to rule out alternative explanations for findings (in qualitative research this could be done by, for example, searching for negative cases/exceptions, feeding back preliminary results to participants, asking a colleague to review the data, or reflexivity).	3.1 Yes, a fairly thorough attempt was made<br><br>3.2 Yes, several steps were taken<br><br>3.3 Yes, minimal few steps were taken<br><br>3.4 No, not at all/ Not stated/Can't tell
**4**	**Were the findings of the study grounded in / supported by**	**Select one:**

	**the data?**	
	Consider whether:<br>- enough data are presented to show how the authors arrived at their findings;<br>- the data presented fit the interpretation/support claims about patterns in data;<br>- the data presented illuminate/illustrate the findings;<br>- quotes are numbered or otherwise identified and the reader can see that they don't just come from one or two people.	4.1 Good grounding/support<br><br>4.2 Fair grounding/support<br><br>4.3 Limited grounding/support
**5**	**Please rate the findings of the study in terms of their breadth and depth.**	**Select one:**
	NB: it may be helpful to consider 'breadth' as the extent of description and 'depth' as the extent to which data has been transformed/analysed).<br><br>*Consider whether:*<br>- a range of issues are covered;<br>- the perspectives of participants are fully explored in terms of breadth (contrast of two or more perspectives) and depth (insight into a single perspective);<br>- richness and complexity has been portrayed (e.g. variation explained, meanings illuminated);<br>- there has been theoretical/conceptual development.	5.1 Good/fair breadth and depth<br><br>5.2 Good /fair depth but very little breadth<br><br>5.3 Good/fair breadth but very little depth<br><br>5.4 Limited breadth or depth
**6**	**To what extent does the study privilege the perspectives and experiences of people with aphasia post-stroke?**	**Select one:**
	Consider whether:<br>- there was a balance between open-ended and fixed response options;<br>- people with aphasia post-stroke were involved in designing the research;<br>- there was a balance between the use of an a priori coding framework and induction in the analysis;<br>- the position of the researchers (did they consider it important to listen to the perspectives of people with aphasia post-stroke ?);<br>- steps were taken to assure confidentiality and put people with aphasia post-stroke at ease.<br>- how were people with aphasia supported to partake in interviews? (e.g. supported conversation techniques; techniques to ensure that significant others did not speak for participants with aphasia)	6.1 A lot<br><br>6.2 Somewhat<br><br>6.3 A little<br><br>6.4 Not at all
**7**	**Overall, what weight would you assign to this study in terms of the reliability/trustworthiness of its findings?**	**Select one:**

	Guidance: Think (mainly) about the answers you have given to questions 1 to 4 above.	7.1 High<br><br>7.2 Medium<br><br>7.3 Low
**8**	**What weight would you assign to this study in terms of the usefulness of its findings for this review?**	**Select one:**
	Think (mainly) about the answers you have given to questions 5 and 6 above and consider:<br>- the match between the study aims and findings and the aims and purpose of the synthesis;<br>- its conceptual depth/explanatory power.	8.1 High<br><br>8.2 Medium<br><br>8.3 Low
