# Supplementary material for: Perspectives of people with aphasia post-stroke towards personal recovery and living successfully: A systematic review and thematic synthesis
Source: PLoS One. 2019 Mar 22;14(3):e0214200. doi: 10.1371/journal.pone.0214200 (PMC6430359; doi:10.1371/journal.pone.0214200)
Supplement: S3 Table — (PDF) [file pone.0214200.s009.pdf]

S7 Data Extraction Instrument

STUDY AND PARTICIPANT CHARACTERISTICS

First author	
Year of publication	
Country	
Research question(s) or objective(s)	
Ethical approval	
Study funding or conflicts of interest	
Data collection	
Qualitative methodology / analysis	
Inclusion criteria	
Exclusion criteria	
Number of participants with aphasia post-stroke<br>Number of men / women<br>Age range and mean age (years)<br>Aphasia type and severity<br>Communication assessments administered<br>Time range and mean time since stroke<br>Living arrangement	

S7 Data Extraction Instrument

Marital status<br>Education / employment status<br>Details of previous therapy / rehabilitation<br>Ethnicity<br>Language(s) spoken<br>Comorbidities	
Number of participants without aphasia	
Number of participants with aphasia of different aetiologies	
Declared study limitations	
Overall conclusion(s) or recommendation(s)	

S7 Data Extraction Instrument

SYNTHESIS DATA

Inclusion and exclusion criteria for data extraction

Research Questions:<br>1. What are people with aphasia post-stroke's perceptions of and attitudes towards recovery and living successfully with aphasia? What do recovery and living successfully with aphasia mean to people with aphasia post-stroke?<br>2. Who or what do people with aphasia post-stroke think has helped them in their recovery and to live successfully with aphasia?<br>3. What do people with aphasia post-stroke think is unhelpful in terms of recovery and living successfully with aphasia?<br>4. What ideas do people with aphasia post-stroke have about what could or should be done to promote recovery and successful living with aphasia?

INCLUDE:	Example
All text / quotes (generally appears in the 'findings/results' section) obtained through interviews and / or focus group discussions with participants with aphasia post-stroke.	'Adrienne expressed this with comments such as, "there's a lot of 'what will it [I] look like at the end [of recovery]', you know?"' (1)
All author comments / interpretations in the 'findings/results' section relating to the experiences, preferences and perspectives of people with aphasia post-stroke, discovered through qualitative interviews and/or focus group discussions.	'*Actively hoping* captured the sense that participants engaged with a future-oriented hope by identifying hopes for the future and working towards hopes' (1)
Any author comments/ interpretations in other sections of the article that (1) relates to the experiences, preferences and perspectives of people with aphasia post-stroke, discovered through qualitative interviews and/or focus group discussions, and (2) are described differently to the main results section.	"It may be that people are more likely to engage in *actively hoping* later in recovery when they are more likely to be active in thinking about the future" (1)
Text appearing in table format including quotes or interpretations will be included in the synthesis only if it does not appear elsewhere within the main body of the text.	

EXCLUDE:	Example
Text / quotes that are not clearly from participants with aphasia post-stroke, or that are clearly from participants without aphasia (e.g. relatives, caregivers, clinicians) and / or people with aphasia of aetiologies other than stroke (e.g. traumatic brain injury).	"A few people were less positive, complaining about therapy being too theoretical or irrelevant to their particular needs. Claire found her therapy patronising and ''stupid'' as her husband Tom explained:<br>Well, I think it was a lost cause. She (the therapist) had a routine … with the cards and all that sort of gear. To my mind that's a bit degrading, putting you back to about grade one or two at school, you know, hat and cat and mat stuff

	…that would frustrate me…" (2)
Author comments/interpretations of data that are not clearly from participants with aphasia post-stroke, or that are clearly from participants without aphasia (e.g. relatives, caregivers, clinicians) and / or people with aphasia of aetiologies other than stroke (e.g. traumatic brain injury).	In the following example it is unclear whether the author is interpreting data from people with aphasia and or their relatives: "People often talked about recovery as unpredictable and non-linear, with periods of positive change and periods of regression. Despite the difficulties of not knowing what would happen, some interviewees remained hopeful for change, even in cases where the aphasia was severe" (2)
Author comments/interpretations of data collected using methods other than qualitative interviews or focus group discussions.	
Author comments/interpretations not directly relating to the experiences, preferences and perspectives of people with aphasia post-stroke (e.g. broader discussion relating to the clinical implications of the key findings and concepts).	"It indicates clinicians should consider hope in people with aphasia after stroke, as it is important to those people and it appears related to common components of the rehabilitation process such as goal setting" (1)
Text that links key concepts and findings with prior literature in the area (generally in discussion section).	"*Actively hoping* may be more difficult because of the uncertainty and limitations associated with recovery and chronic illness (Becker & Kaufman, 1995; Carlsson, Moller, & Blomstrand, 2009; Kim et al., 2006; Rittman et al., 2004)" (1)
Meta-comments regarding the study methodology and its possible impact on the key findings and concepts (generally in discussion section).	"The questions asked influenced the responses with different questions identifying different aspects of the experience of hope" (1)
Tables (commonly appearing in the results/findings sections) that list key factors, concepts, themes which are already listed and described within the main body of the text; and which do not contain quotes or interpretations that appear elsewhere in the main body of the text. This is to avoid duplication and unnecessary lists within the synthesis data.	"Box 1:<br>perceived engagement in participation<br>defined by:<br>. Being involved<br>. Not being burdensome<br>. Knowing what is going on<br>. Taking part<br>. Being respected" (3)

1. Bright FAS, Kayes NM, McCann CM, McPherson KM. Hope in people with aphasia. Aphasiology. 2013;27(1):41-58.
2. Hersh D. How do people with aphasia view their discharge from therapy? Aphasiology. 2009;23(3):331-50.
3. Dalemans RJ, de Witte L, Wade D, van den Heuvel W. Social participation through the eyes of people with aphasia. Int J Lang Commun Disord. 2010;45(5):537-50.
