# Supplementary material for: Perspectives of people with aphasia post-stroke towards personal recovery and living successfully: A systematic review and thematic synthesis
Source: PLoS One. 2019 Mar 22;14(3):e0214200. doi: 10.1371/journal.pone.0214200 (PMC6430359; doi:10.1371/journal.pone.0214200)
Supplement: S4 Table — (PDF) [file pone.0214200.s010.pdf]

S8 Summary of included studies

	Reference	Country	Population	No. participants with aphasia post-stroke	Sex: m/f	Mean age (years)	Mean time since stroke (years)	Data collection	Methodology / Analysis	Research objective(s)
1	Armstrong E, Hersh D, Hayward C, Fraser J, Brown M. Living with aphasia: three Indigenous Australian stories. Int J Speech Lang Pathol. 2012;14(3):271-80.	Australia	Aboriginal Australian adults with acquired communication disorders post-stroke and their family members or community workers	3	3m	54.3, range: 47-63	15.5, range: 0.5-29 years	Semi-structured interviews	Thematic	To present the stories of living with aphasia for three Indigenous Australian men living in Perth, Western Australia.
2	Armstrong E, Hersh D, Hayward C, Fraser J. Communication disorders after stroke in Aboriginal Australians. Disabil Rehabil. 2015;37(16-17):1462-9.	Australia	13 Aboriginal Australian adults with acquired communication disorders post-stroke and their family members or community workers	7	4m, 3w	49.6, range: 30-63	9.6, range: 0.5-29	Semi-structured interviews (linked with Armstrong et al 2012).	Thematic	To explore Aboriginal Australians' experiences of acquired communication disorders in terms of functional and personal consequences as well as their experiences of SLT and other services received after stroke
3	Berg K, Askim T, Balandin S, Armstrong E, Rise MB. Experiences of participation in goal setting for people with stroke-induced aphasia in	Norway	Adults with aphasia post-stroke	15	7m, 8w	61, range: 43-74	Range 4 months - 9.7 years	Interviews	Phenomenology - Systematic Text Condensation	To explore how PWA experienced client participation during the process of goal setting and clinical decision making in language rehabilitation

S8 Summary of included studies

	Norway. A qualitative study. Disabil Rehabil. 2016:1-9.									
4	Bright FAS, Kayes NM, McCann CM, McPherson KM. Hope in people with aphasia. Aphasiology. 2013;27(1):41-58.	New Zealand	Adults with aphasia post-stroke	5	3m, 2w	50, range: 41-62	2.8, range: 2-5 months	Semi-structured interviews	Interpretive Description incorporating multiple analytic strategies including coding, constant comparison, narrative construction, thematic analysis, diagramming, memos.	To examine how PWA experience hope in the post-acute period of rehabilitation and what factors appear to influence a person's experience of hope.
5	Brown K, Worrall L, Davidson B, Howe T. Snapshots of success: An insider perspective on living successfully with aphasia. Aphasiology. 2010;24(10):1267-95.	Australia	Adults with aphasia post-stroke	25	13m, 12w	63.2 ± 12.2	6 ± 5.2	Semi-structured interviews and participant-generated photography	Interpretative Phenomenological Analysis	To explore, from the perspectives of PWA, the meaning of living successfully with aphasia
6	Brown K, Davidson B, Worrall L, Howe T. "Making a good time": the role of friendship in living successfully with aphasia. Int J Speech Lang	Australia	Adults with aphasia post-stroke	25	13m, 12w	63.2 ± 12.2	6 ± 5.2	Analysis of data collected in Brown et al (2010)	Thematic	To explore the perspectives of PWA on the role of friendship in living successfully with aphasia.

S8 Summary of included studies

	Pathol. 2013;15(2):165-75.									
7	Cruice M, Worrall L, Hickson L. Perspectives of quality of life by people with aphasia and their family: suggestions for successful living. Top Stroke Rehabil. 2006;13(1):14-24.	Australia	Adults with aphasia post-stroke	4	4w	77, range: 68-81d	range: 13 months - 3.5 years	Structured interviews	Case study	To describe the perspectives of 4 older individuals with stroke and aphasia on what gives them quality in their lives
8	Cruice M, Hill R, Worrall L, Hickson L. Conceptualising quality of life for older people with aphasia. Aphasiology. 2010;24(3):327-47.	Australia	Adults with aphasia post-stroke	30	14m, 16w	70.7, range: 57-88	3.4 ± 2.1	Structured interviews (linked with Cruice et al 2006)	Content analysis	To conceptualise how older people with chronic aphasia who are living in the community describe their quality of life in terms of contributors and detractors.
9	Dalemans RJ, de Witte L, Wade D, van den Heuvel W. Social participation through the eyes of people with aphasia. Int J Lang Commun Disord. 2010;45(5):537-50.	The Netherlands	Adults with aphasia post-stroke and their caregivers	13	7m, 6w	57.4, range: 45-71	5.1, range: 1.4-11	Semi-structured interviews, diary, focus groups	Open coding and categorisation	To explore how PWA perceive participation in society and to investigate influencing factors
10	Dietz A, Thiessen	United	Adults with	3	2m, 1w	56.3, range:	7.3, range:	Semi-	Phenomenology,	To explore the social role

S8 Summary of included studies

	A, Griffith J, Peterson A, Sawyer E, McKelvey M. The renegotiation of social roles in chronic aphasia: Finding a voice through AAC. Aphasiology. 2013;27(3):309-25.	States	aphasia post-stroke and their relatives / friends			41-85	2-15	structured interviews	inductive approach	changes experienced by PWA, to understand the use of communication strategies when attempting to reclaim previous social roles and to determine whether discrepancies existed between the accounts of PWA and their potential proxies.
11	Doughty Horn EA. Identifying and addressing grief and loss issues in a person with aphasia: A single-case study. Journal of Counseling & Development. 2016;94(2):225-34.	United States	Adults with aphasia post-stroke	1	1w	Not available	Not available	Semi-structured interviews	Phenomenological reduction and coding	To provide a rich description of one individual's experiences with aphasia to help counsellors better conceptualise grief and loss issues in aphasia and to inform future counselling relationships
12	Grohn B, Worrall LE, Simmons-Mackie N, Brown K. The first 3-months post-stroke: what facilitates successfully living with aphasia? Int J Speech Lang Pathol. 2012;14(4):390-400.	Australia	Adults with aphasia	15	8m, 7w	66.3 ± 13.6	All within 3 months +/- 2 weeks	Semi-structured interviews	Thematic	To explore the perspectives of PWA on factors that facilitate successful living during the first 3 months post-onset.
13	Grohn B, Worrall	Australia	Adults with	15	8m, 7w	66.3 ± 13.6	Interviews	Semi-	Phenomenology -	To describe the insider's

S8 Summary of included studies

	L, Simmons-Mackie N, Hudson K. Living successfully with aphasia during the first year post-stroke: A longitudinal qualitative study. Aphasiology. 2014;28(12):1405-25.		aphasia post-stroke				conducted at 3, 6, 9 and 12 months post-stroke	structured interviews (continuation of Grohn et al 2012)	Thematic	perspective of what is important to living successfully with aphasia and changes that occur throughout the first year post-stroke.
14	Hemsley B, Werninck M, Worrall L. "That really shouldn't have happened": People with aphasia and their spouses narrate adverse events in hospital. Aphasiology. 2013;27(6):706-22.	Australia	Adults with aphasia (including 1 with Primary Progressive Aphasia) and their spouses	10	8m, 2w	68.4, range: 64-71	5.4, range: 1-11	Interviews	Narrative	To describe and understand the adverse events experienced by PWA and their spouses in hospital
15	Hersh D. How do people with aphasia view their discharge from therapy? Aphasiology. 2009;23(3):331-50.	Australia	Adults with aphasia and their family members	21	13m, 8w	Range: 44-85	Not available	Interviews	Grounded theory	To explore how PWA view their discharge from therapy.
16	Hersh D. Hopeless, sorry, hopeless: Co-	Australia	Adults with aphasia post-stroke	2	2w	34-61	4-8 years	Secondary analysis of Worrall et al	Narrative	To consider the knowledge, skills and attitudes underpinning co-

	constructing narratives of care with people who have aphasia post-stroke. Topics in Language Disorders. 2015;35(3):219-37.							(2011) data collected (by first author) collected using semi-structured interviews		construction in the context of health care and to use examples from interviews with 2 PWA to illustrate narrative competence, the richness of their stories, and what lessons might be learned.
17	Hjelmblink F, Bernsten CB, Uvhagen H, Kunkel S, Holmström I. Understanding the meaning of rehabilitation to an aphasic patient through phenomenological analysis – a case study. International Journal of Qualitative Studies on Health and Well-being. 2007;2:93-100.	Sweden	Adults with aphasia post-stroke	1	1m	58 years	1st interview 3 months; 2nd interview 12 months	Interviews	Phenomenology - Empirical Phenomenological & Psychological method (EPP)	To reveal the meaning of rehabilitation to a PWA, such as it was lived by him.
18	Howe TJ, Worrall LE, Hickson LMH. Interviews with people with aphasia: Environmental factors that influence their community	Australia	Adults with aphasia post-stroke	25	15m, 10w	62.2 ± 10.7	5.6 ± 2.9	Semi-structured interviews	Content analysis	To explore the environmental factors that hinder or support the community participation of PWA.

	participation. Aphasiology. 2008;22(10):1092-120.									
19	Jones F, Mandy A, Partridge C. Reasons for recovery after stroke: A perspective based on personal experience. Disabil Rehabil. 2008;30(7):507-16.	United Kingdom	10 people with stroke	3	2m, 1w	59.7, range: 56-64	7.5 months, range: 6 weeks - 13 months	Interviews	Content analysis	To learn about individual beliefs and personal strategies used to support the period of recovery after stroke
20	Le Dorze G, Brassard C. A description of the consequences of aphasia on aphasic persons and their relatives and friends, based on the WHO model of chronic diseases. Aphasiology. 1995;9(3):239-55.	Canada	Adults with aphasia post-stroke and their relatives / friends	9	5m, 4w	54.2, range: 41-69	6.6, range: 2-14	Semi-structured interviews	Constant comparison and deductive analysis	To describe the experience and consequences of aphasia from the perspectives of PWA and their friends and relatives and code according to the WHO ICIDH
21	Le Dorze G, Salois-Bellerose É, Alepins M, Croteau C, Hallé M-C. A description of the personal and environmental determinants of participation	Canada	Adults with aphasia post-stroke	17	12m, 5w	65.7 ± 7.5	5.7, range: 2-18	Focus groups	Content analysis	To explore the factors that facilitate or hinder participation according to people who live with aphasia.

	several years post-stroke according to the views of people who have aphasia. Aphasiology. 2014;28(4):421-39.									
22	MacKenzie C, Bennett A, Cairney M. Active citizenship and acquired neurological communication difficulty. Disabil Rehabil. 2011;33(3):187-94.	United Kingdom	6 adults with communication support needs (CSN) from acquired neurological lesions including post-stroke aphasia and dysarthria, Parkinson's Disease and dysarthria and Dementia.	3	1m, 2w	range: 54-78 (all participants)	range: 3-22 years (all participants)	Interviews	Content analysis	To explore the experience of civically engaged adults with CSN in terms of motivations, barriers and changes which might facilitate more effective civic participation of people with CSN.
23	Mc Menamin R, Tierney E, Mac Farlane A. Addressing the long-term impacts of aphasia: how far does the Conversation Partner Programme go? Aphasiology. 2015;29(8):889-913.	Ireland	Adults with aphasia post-stroke	5	4m, 1w	73, range 60-85	5.4, range 3-10	Participatory Learning and Action (PLA) data co-generation sessions	Thematic	To describe participants' insider (emic) experiences of (1) aphasia and (2) a Conversation Partner Programme.
24	McLellan KM, McCann CM, Worrall LE,	New Zealand	Māori adults with aphasia and their	11	4m, 7w	Min age range: 50-59;	Range <2 - 10+ years	Semi-structured interviews	Interpretive description, Kaupapa Māori	To describe and interpret the experiences of Māori with aphasia and their whānau

	Harwood MLN. “For Māori, language is precious. And without it we are a bit lost”: Māori experiences of aphasia. Aphasiology. 2013;28(4):453-70.		whānau (extended family)			Max age range: 70-79			research (KMR) approach	(extended family), to inform service delivery for this population.
25	Morris K, Ferguson A, Worrall L. A qualitative study of legal and social justice needs for people with aphasia. Int J Speech Lang Pathol. 2014;16(6):541-51.	Australia	Adults with aphasia post-stroke	50	24m, 26w	63.9 ± 10.8, range 32-85	4.6 ± 3.6	Secondary analysis of Worrall et al (2011) data collected using semi-structured interviews	Content analysis	To provide first step toward further research into the prevalence of legal and access to justice issues for PWA and to provide preliminary information to inform the development of processes to ensure access to legal information and services for PWA.
26	Niemi T, Johansson U. The lived experience of engaging in everyday occupations in persons with mild to moderate aphasia. Disabil Rehabil. 2013;35(21):1828-34.	Finland	Adults with aphasia post-stroke	6	3m, 3w	57.7, range 46-75	2.5 years, range 1-4 years	Semi-structured interviews	Phenomenology - Empirical Phenomenological & Psychological method (EPP)	To describe and explore how PWA following stroke experience engaging in everyday occupations.
27	Northcott S, Hilari K. Why do people lose their friends	United Kingdom	29 adults with stroke	10	6m, 4w	57, range 48-74	All 8-15 months post-stroke.	Semi-structured interviews	Framework	To explore why people lose contact with their friends, whether there are any

S8 Summary of included studies

	after a stroke? Int J Lang Commun Disord. 2011;46(5):524-34.									protective factors and how friendship loss and change is perceived by the individual.
28	Parr S. Psychosocial aspects of aphasia: Whose perspectives? Folia Phoniatrica Et Logopaedica. 2001;53(5):266-88.	United Kingdom	Adults with aphasia post-stroke	50	28m, 22w	Minimum age range: <45; Maximum age range: 75+.	7.4, range slightly less than 5 years - 22years	Semi-structured interviews	Framework	To summarise some key features / findings of an earlier qualitative study (Parr et al 1997) and to explore the 'meaning' of aphasia from the insider perspective in terms of its long-term consequences and significance in people's lives.
29	Pearl G, Sage K, Young A. Involvement in volunteering: an exploration of the personal experience of people with aphasia. Disabil Rehabil. 2011;33(19-20):1805-21.	United Kingdom	Adults with aphasia post-stroke	15	Interviews: 6m, 4w; Focus groups: 4m, 1w.	Minimum age range: 30-45; Maximum age range: 60+.	Interviews: 5.6, range 3-12<br>Focus groups: 4.6, range 1-10	Semi-structured interviews and focus groups	Thematic	To identify the perceptions and experiences of PWA towards the effects of participating in volunteering activity and the barriers and facilitators to effective participation.
30	Tomkins B, Siyambalapitiya S, Worrall L. What do people with aphasia think about their health care? Factors influencing satisfaction and dissatisfaction. Aphasiology. 2013;27(8):972-	Australia	Adults with aphasia post-stroke	50	24m, 26w	63.9 ± 10.8, range 32-85	4.6 ± 3.6	Secondary analysis of Worrall et al (2011) data collected using semi-structured interviews	Content analysis	To explore the factors influencing the satisfaction and dissatisfaction of PWA with regards to their healthcare

	91.									
31	Worrall L, Sherratt S, Rogers P, Howe T, Hersh D, Ferguson A, et al. What people with aphasia want: Their goals according to the ICF. Aphasiology. 2011;25(3).	Australia	Adults with aphasia post-stroke	50	24m, 26w	63.9 ± 10.8, range 32-85	4.6 ± 3.6	Semi-structured interviews	Content analysis	To describe the goals of PWA and to code the goals according to the ICF.
