# Supplementary material for: Perspectives of people with aphasia post-stroke towards personal recovery and living successfully: A systematic review and thematic synthesis
Source: PLoS One. 2019 Mar 22;14(3):e0214200. doi: 10.1371/journal.pone.0214200 (PMC6430359; doi:10.1371/journal.pone.0214200)
Supplement: S5 Table — (PDF) [file pone.0214200.s011.pdf]

S9 Summary of critical appraisal

		Were steps taken to increase rigour in the sampling?	Were steps taken to increase rigour in the data collected?	Were steps taken to increase rigour in the data analysis?	Were the findings of the study grounded in / supported by the data?	Please rate the findings of the study in terms of their breadth and depth.	To what extent does the study privilege the perspectives and experiences of people with aphasia post-stroke?	Overall, what weight would you assign to this study in terms of the reliability/ trustworth-iness of its findings?	What weight would you assign to this study in terms of the usefulness of its findings for this review?
		1.1 Yes, a fairly thorough attempt was made<br><br>1.2 Yes, several steps were taken<br><br>1.3 Yes, minimal few steps were taken<br><br>1.4 No, not at all/ Not stated/Can't tell	2.1 Yes, a fairly thorough attempt was made<br><br>2.2 Yes, several steps were taken<br><br>2.3 Yes, minimal few steps were taken<br><br>2.4 No, not at all/ Not stated/Can't tell	3.1 Yes, a fairly thorough attempt was made<br><br>3.2 Yes, several steps were taken<br><br>3.3 Yes, minimal few steps were taken<br><br>3.4 No, not at all/ Not stated/Can't tell	4.1 Good grounding/ support<br><br>4.2 Fair grounding/support<br><br>4.3 Limited grounding/ support	5.1 Good/fair breadth and depth<br><br>5.2 Good /fair depth but very little breadth<br><br>5.3 Good/fair breadth but very little depth<br><br>5.4 Limited breadth or depth	6.1 A lot<br><br>6.2 Somewhat<br><br>6.3 A little<br><br>6.4 Not at all	7.1 High<br><br>7.2 Medium<br><br>7.3 Low	8.1 High<br><br>8.2 Medium<br><br>8.3 Low
1	Armstrong E, Hersh D, Hayward C, Fraser J, Brown M. Living with aphasia: three Indigenous Australian stories. Int J	1.2	2.2	3.2	4.1	5.2	6.2	7.2	8.1

S9 Summary of critical appraisal

	Speech Lang Pathol. 2012;14(3):271-80.								
2	Armstrong E, Hersh D, Hayward C, Fraser J. Communication disorders after stroke in Aboriginal Australians. Disabil Rehabil. 2015;37(16-17):1462-9.	1.1	2.2	3.2	4.1	5.1	6.2	7.2	8.1
3	Berg K, Askim T, Balandin S, Armstrong E, Rise MB. Experiences of participation in goal setting for people with stroke-induced aphasia in Norway. A qualitative study. Disabil Rehabil. 2016:1-9.	1.1	2.2	3.1	4.1	5.2	6.2	7.2	8.2
4	Bright FAS, Kayes NM, McCann CM, McPherson KM. Hope in people with aphasia. Aphasiology. 2013;27(1):41-58.	1.2	2.2	3.1	4.1	5.2	6.2	7.2	8.1
5	Brown K, Worrall L, Davidson B, Howe T. Snapshots of success: An insider perspective on living successfully with aphasia. Aphasiology. 2010;24(10):1267-95.	1.1	2.2	3.1	4.2	5.1	6.1	7.1	8.1
6	Brown K, Davidson B, Worrall L, Howe T.	1.1	2.2	3.2	4.2	5.1	6.1	7.2	8.1

S9 Summary of critical appraisal

	"Making a good time": the role of friendship in living successfully with aphasia. Int J Speech Lang Pathol. 2013;15(2):165-75.								
7	Cruice M, Worrall L, Hickson L. Perspectives of quality of life by people with aphasia and their family: suggestions for successful living. Top Stroke Rehabil. 2006;13(1):14-24.	1.3	2.2	3.4	4.1	5.1	6.2	7.3	8.1
8	Cruice M, Hill R, Worrall L, Hickson L. Conceptualising quality of life for older people with aphasia. Aphasiology. 2010;24(3):327-47.	1.2	2.2	3.2	4.2	5.3	6.2	7.2	8.2
9	Dalemans RJ, de Witte L, Wade D, van den Heuvel W. Social participation through the eyes of people with aphasia. Int J Lang Commun Disord. 2010;45(5):537-50.	1.1	2.2	3.2	4.1	5.2	6.1	7.1	8.1
10	Dietz A, Thiessen A, Griffith J, Peterson A, Sawyer E, McKelvey M. The renegotiation of social roles in chronic aphasia: Finding a voice through AAC.	1.4	2.3	3.3	4.2	5.3	6.3	7.3	8.3

S9 Summary of critical appraisal

	Aphasiology. 2013;27(3):309-25.								
11	Doughty Horn EA. Identifying and addressing grief and loss issues in a person with aphasia: A single-case study. Journal of Counseling & Development. 2016;94(2):225-34.	1.2	2.2	3.1	4.1	5.2	6.1	7.2	8.1
12	Grohn B, Worrall LE, Simmons-Mackie N, Brown K. The first 3-months post-stroke: what facilitates successfully living with aphasia? Int J Speech Lang Pathol. 2012;14(4):390-400.	1.1	2.2	3.2	4.2	5.1	6.2	7.2	8.1
13	Grohn B, Worrall L, Simmons-Mackie N, Hudson K. Living successfully with aphasia during the first year post-stroke: A longitudinal qualitative study. Aphasiology. 2014;28(12):1405-25.	1.1	2.2	3.1	4.2	5.1	6.2	7.2	8.1
14	Hemsley B, Werninck M, Worrall L. “That really shouldn't have happened”: People with aphasia and their spouses	1.3	2.2	3.2	4.2	5.3	6.2	7.2	8.3

	narrate adverse events in hospital. Aphasiology. 2013;27(6):706-22.								
15	Hersh D. How do people with aphasia view their discharge from therapy? Aphasiology. 2009;23(3):331-50.	1.2	2.2	3.2	4.1	5.1	6.2	7.2	8.1
16	Hersh D. Hopeless, sorry, hopeless: Co-constructing narratives of care with people who have aphasia post-stroke. Topics in Language Disorders. 2015;35(3):219-37.	1.1	2.2	3.1	4.1	5.2	6.1	7.1	8.1
17	Hjelmblink F, Bernsten CB, Uvhagen H, Kunkel S, Holmström I. Understanding the meaning of rehabilitation to an aphasic patient through phenomenological analysis – a case study. International Journal of Qualitative Studies on Health and Well-being. 2007;2:93-100.	1.4	2.3	3.1	4.1	5.2	6.3	7.2	8.2
18	Howe TJ, Worrall LE, Hickson LMH. Interviews with people with aphasia: Environmental factors that influence their	1.1	2.2	3.1	4.2	5.3	6.1	7.2	8.2

S9 Summary of critical appraisal

	community participation. Aphasiology. 2008;22(10):1092-120.								
19	Jones F, Mandy A, Partridge C. Reasons for recovery after stroke: A perspective based on personal experience. Disabil Rehabil. 2008;30(7):507-16.	1.1	2.2	3.1	4.1	5.1	6.3	7.1	8.3
20	Le Dorze G, Brassard C. A description of the consequences of aphasia on aphasic persons and their relatives and friends, based on the WHO model of chronic diseases. Aphasiology. 1995;9(3):239-55.	1.2	2.2	3.2	4.2	5.3	6.3	7.2	8.3
21	Le Dorze G, Salois-Bellerose É, Alepins M, Croteau C, Hallé M-C. A description of the personal and environmental determinants of participation several years post-stroke according to the views of people who have aphasia. Aphasiology. 2014;28(4):421-39.	1.2	2.2	3.2	4.2	5.3	6.2	7.2	8.2
22	MacKenzie C, Bennett A, Cairney M. Active	1.3	2.3	3.2	4.2	5.3	6.3	7.3	8.3

	citizenship and acquired neurological communication difficulty. Disabil Rehabil. 2011;33(3):187-94.								
23	Mc Menamin R, Tierney E, Mac Farlane A. Addressing the long-term impacts of aphasia: how far does the Conversation Partner Programme go? Aphasiology. 2015;29(8):889-913.	1.1	2.1	3.1	4.1	5.1	6.1	7.1	8.2
24	McLellan KM, McCann CM, Worrall LE, Harwood MLN. "For Māori, language is precious. And without it we are a bit lost": Māori experiences of aphasia. Aphasiology. 2013;28(4):453-70.	1.1	2.2	3.1	4.1	5.1	6.3	7.1	8.3
25	Morris K, Ferguson A, Worrall L. A qualitative study of legal and social justice needs for people with aphasia. Int J Speech Lang Pathol. 2014;16(6):541-51.	1.2	2.2	3.3	4.2	5.3	6.2	7.2	8.3
26	Niemi T, Johansson U. The lived experience of engaging in everyday occupations in persons with mild to moderate aphasia. Disabil Rehabil.	1.3	2.2	3.2	4.3	5.2	6.2	7.2	8.2

S9 Summary of critical appraisal

	2013;35(21):1828-34.								
27	Northcott S, Hilari K. Why do people lose their friends after a stroke? Int J Lang Commun Disord. 2011;46(5):524-34.	1.1	2.1	3.1	4.1	5.1	6.3	7.1	8.3
28	Parr S. Psychosocial aspects of aphasia: Whose perspectives? Folia Phoniatrica Et Logopaedica. 2001;53(5):266-88.	1.1	2.1	3.1	4.1	5.1	6.1	7.1	8.1
29	Pearl G, Sage K, Young A. Involvement in volunteering: an exploration of the personal experience of people with aphasia. Disabil Rehabil. 2011;33(19-20):1805-21.	1.1	2.1	3.1	4.1	5.1	6.1	7.1	8.1
30	Tomkins B, Siyambalapitiya S, Worrall L. What do people with aphasia think about their health care? Factors influencing satisfaction and dissatisfaction. Aphasiology. 2013;27(8):972-91.	1.2	2.2	3.2	4.1	5.1	6.1	7.1	8.1

S9 Summary of critical appraisal

31	Worrall L, Sherratt S, Rogers P, Howe T, Hersh D, Ferguson A, et al. What people with aphasia want: Their goals according to the ICF. Aphasiology. 2011;25(3).	1.2	2.2	3.3	4.2	5.3	6.2	7.2	8.2
